# Mathematical Modeling of a Supramolecular Assembly for Pyrophosphate Sensing

**DOI:** 10.3389/fchem.2021.759714

**Published:** 2021-12-21

**Authors:** Fereshteh Emami, Hamid Abdollahi, Tsyuoshi Minami, Ben Peco, Sean Reliford

**Affiliations:** ^1^ Department of Chemistry and Physics, Southeastern Louisiana University, Hammond, LA, United States; ^2^ Department of Chemistry, Institute for Advanced Studies in Basic Sciences, Zanjan, Iran; ^3^ Institute of Industrial Science, The University of Tokyo, Meguro-ku, Japan

**Keywords:** mathematical modeling, supramolecules, molecular sensing, pyrophosphate, intertwined equilibria, thermodynamic parameter fitting, enthalpy, entropy

## Abstract

The power of sensing molecules is often characterized in part by determining their thermodynamic/dynamic properties, in particular the binding constant of a guest to a host. In many studies, traditional nonlinear regression analysis has been used to determine the binding constants, which cannot be applied to complex systems and limits the reliability of such calculations. Supramolecular sensor systems include many interactions that make such chemical systems complicated. The challenges in creating sensing molecules can be significantly decreased through the availability of detailed mathematical models of such systems. Here, we propose uncovering accurate thermodynamic parameters of chemical reactions using better-defined mathematical modeling-fitting analysis is the key to understanding molecular assemblies and developing new bio/sensing agents. The supramolecular example we chose for this investigation is a self-assembled sensor consists of a synthesized receptor, DPA (DPA = dipicolylamine)-appended phenylboronic acid (**1**) in combination with Zn^2+^(**1**.Zn) that forms various assemblies with a fluorophore like alizarin red S (ARS). The self-assemblies can detect multi-phosphates like pyrophosphate (PPi) in aqueous solutions. We developed a mathematical model for the simultaneous quantitative analysis of twenty-seven intertwined interactions and reactions between the sensor (**1**.Zn-ARS) and the target (PPi) for the first time, relying on the Newton-Raphson algorithm. Through analyzing simulated potentiometric titration data, we describe the concurrent determination of thermodynamic parameters of the different guest-host bindings. Various values of temperatures, initial concentrations, and starting pHs were considered to predict the required measurement conditions for thermodynamic studies. Accordingly, we determined the species concentrations of different host-guest bindings in a generalized way. This way, the binding capabilities of a set of species can be quantitatively examined to systematically measure the power of the sensing system. This study shows analyzing supramolecular self-assemblies with solid mathematical models has a high potential for a better understanding of molecular interactions within complex chemical networks and developing new sensors with better sensing effects for bio-purposes.

## 1 Introduction

The capability to precisely detect a target system and monitor its variation over time is highly desirable in various fields, including biological studies, diagnosis, and quality control. Some sensor-target combinations include equilibrium, kinetic, or intertwined equilibrium-kinetic chemical interactions like supramolecular sensors (Johnson-Buck et al., 2015). Other sensor interactions with targets may trigger chemical, electrical, and mechanical changes like hydrogel sensors (Gladman et al., 2016). Supramolecular chemistry is an emergent research field taking its roots in chemistry. Its combination with biology opens a new direction in the study of life and its origin (Vicens and Vicens, 2011). The last several years have seen substantial growth in the field, and many detection efforts have focused on the use of fluorescent supramolecular sensors in cells and tissues (Lim et al., 2005; Palmer and Tsien, 2006; Que et al., 2008; Wong et al., 2009). In many chemical and biological processes, including molecular self-assembly and protein-protein or protein-substrate recognition, noncovalent interactions are critically important (Mugridge, 2010). Drawing inspiration from such natural systems, supramolecular chemists seek to use these weak, nonbonding interactions to mediate the formation and reactivity of host-guest complexes. Sensing applications of supramolecular chemistry rely on exploiting the forces involved in the formation of non-covalent “host-guest” complexes. In all cases, having a binding site that is highly specific for an analyte “guest” in a measurable form is the critical key for the “host” molecules (Lehn, 1994; Goral et al., 2001; Boul et al., 2005; Pérez-Fuertes et al., 2006, 2007; Ayme et al., 2012; You et al., 2015). The continued development of improved sensors depends on achieving a thorough understanding of the underlying chemical properties of the available constructs.

The determination of accurate binding constants is an important prerequisite for the development of many host-guest complexes, which range from sensing to drug discovery and development (Giri et al., 2011; Meier and Beeren, 2014). Unfortunately, in current analyses of supramolecular self-assemblies, no efficient mathematical modeling is applied to obtain these constants. Regular 1:1 or 1:2 complexes, an indicator displacement assay, or an enantioselective indicator displacement assay are usually considered, and the apparent binding constants are determined (Connors, 1987; Hargrove et al., 2010; Sheff et al., 2011). While many assemblies are produced from the reactions/interactions among different species.

The pH has a crucial role in most chemical processes, and many reactions in aqueous solutions show a strong pH dependence (Maeder et al., 2003; Wilkins, 1991). Protonation equilibria are particularly complex with multidentate ligands. Several differently protonated forms of the ligand usually coexist in solution; all of these forms show different reactivities towards each other. As a result, the observed equilibria are strongly pH dependent. Therefore, pH-metric is a practical tool to gain insight into the reactivities of the supramolecular sensors. In this study, we use a simple potentiometric pH method to simultaneously determine thermodynamic constants of the interactions among a self-assembled sensor prepared from Zn^II^–DPA (DPA = dipicolylamine)-attached phenylboronic acid (**1**·Zn) and catechol-type dye, alizarin red S (ARS) towards pyrophosphate (PPi). We have previously used these self-assemblies to detect oligophosphates in aqueous solutions (Minami et al., 2016). We considered an indicator displacement assay and determined the apparent binding constants of ARS toward **1**.Zn and phosphates toward the sensor ARS-**1**.Zn. However, different analytical measurements displayed the presence of multiple intertwined equilibria, including twenty-seven equilibrium interactions and reactions. A mixture of such complicated combinations makes it impossible for the simple curve fitting to reproduce the observed progression. We chose 1.Zn-ARS-PPi sensing system because, according to our previous studies, this self-assembly includes many interactions that make it very complicated. So, it is a great candidate to walk the readers through the mathematical equations. Although, this study can be generalized to any other self-assembly examples.

Here, to address this problem and avoid as many assumptions as possible, we develop iterative methods that allow for the rigorous modeling of binding equations with the help of the Newton-Raphson algorithm in MATLAB, describing the complete molecular behaviors. We highlight how the extraction of binding data from a network of equilibria with the help of well-defined mathematical modeling led to having equilibria information of all recognized species to study interactions in chemical systems. This method should be generally applicable so long as the supramolecular system is pH-dependent.

## 2 Computational Details

We start with an introduction to the theory of equilibria in solution, the law of mass action, the notations required for the quantitative description of the interactions, which can be rather complex, particularly in aqueous solutions. This is followed by a discussion of the types of measurements that can be carried out and the nature of the data that are delivered. Next, we introduce the computational methods are required for the analysis of the measurements. To give the reader the possibility to apply all the above concepts, we use three practical examples: the potentiometric investigation of the interactions of 1.Zn-ARS-PPi. A suite of MATLAB programs that perform the analysis of the above data sets is available from the authors.

To introduce the mathematical modeling concept, consider a species formed by only three components A , B , and C (Eq. 1).

$$aA+bB+cC⇄A_{a}B_{b}C_{c}\;\;\;\;\;\beta_{abc}=\frac{\left[A_{a}B_{b}C_{c}\right]}{{\left[A\right]}^{a}{\left[B\right]}^{b}{\left[C\right]}^{c}}\;$$ (1)

where $a,b,c=1,2,3\ldots \text{and }A_{a}B_{b}C_{c}$ is the product of sensor and target combination. $\beta_{abc}$ is the formation constant of the species $A_{a}B_{b}C_{c}$ . The definition of the stabilities of all species as a function of the component concentrations *via* the $\beta_{abc}$ values is consistent and allows the development of general, compact, and thus fast computer programs for data analysis.

Computer programs written for the analysis of measurements for equilibrium investigations contain two parts that require specific attention. The more obvious one is the algorithm for parameter fitting. Its task is to determine the optimal values for the parameters for a given measurement and model. In a titration experiment, the parameters to be fitted are usually the formation constants and, in the case of a spectrophotometric titration, additionally the molar absorption spectra of all-absorbing species. The other important part is computing all species concentrations for a given set of total component concentrations and formation constants. This calculation has to be performed for the solution after each addition of reagent during the complete titration. This second task forms the core of the data fitting and, therefore, we will discuss it first.

### 2.1 The Newton-Raphson Algorithm

The quantitative analysis of chemical equilibria is achieved using the Newton-Raphson algorithm that computes the species concentrations for a given set of formation constants and total concentrations of the components ${\left[A\right]}_{tot}$ , ${\left[B\right]}_{tot}$ , and ${\left[C\right]}_{tot}$ . The components are the basic units that interact with each other to form the species. The basis for the computations is the law of conservation of mass. At equilibrium, different species defined in the model reach a certain concentration that obeys the mass law. It means the law of mass action relates the species concentrations to the total concentrations of the components. For instance, the concentration summation of A existing in different species forms has to equal the known total concentration ${\left[A\right]}_{tot}$ .

The quantitative relationship between the concentrations of free components and species is defined by the formation constants:

$$\left[A_{a}B_{b}C_{c}\right]=\beta_{abc}{\left[A\right]}^{a}{\left[B\right]}^{b}{\left[C\right]}^{c}$$ (2)

Each species concentration is computed from the formation constants and the free component concentrations as given in Eq. 2 for the general equilibrium (Eq. 1. For each of the components, A , B , and C , we can write the following equations:

$$\begin{array}{l} {\left[A\right]}_{tot-calc}=\left[A\right]+a\left[A_{a}B_{b}C_{c}\right]\\ {\left[B\right]}_{tot-calc}=\left[B\right]+b\left[A_{a}B_{b}C_{c}\right]\\ {\left[C\right]}_{tot-calc}=\left[C\right]+c\left[A_{a}B_{b}C_{c}\right]\; \end{array}$$ (3)

In a titration process, ${\left[A\right]}_{tot}$ , ${\left[B\right]}_{tot}$ , and ${\left[C\right]}_{tot}$ are the known independent variables and are computed from the reagent solutions and dilutions occurring during the titration. The total concentrations of the components are stored in the matrix **C**_**tot**_. These total concentrations have to equal the sums over all appropriately weighted species concentrations, Eq. 3. The differences between the calculated and known total concentrations are collected in the vector diff . The goal of the algorithm is to determine the free component concentrations such that the differences diff are zero.

$$\begin{array}{l} \mathrm{dif}f_{A}={\left[A\right]}_{tot}-{\left[A\right]}_{tot-calc}\\ \mathrm{dif}f_{B}={\left[B\right]}_{tot}-{\left[B\right]}_{tot-calc}\\ \mathrm{dif}f_{C}={\left[C\right]}_{tot}-{\left[C\right]}_{tot-calc}\; \end{array}$$ (4)

By substituting Eq. 2 in Eq. 3, each total component concentration can be calculated as below and stored in the matrix ${\left[Component\right]}_{tot-calc}$ :

$$\begin{array}{l} {\left[A\right]}_{tot-calc}=\overset{nspec}{\underset{i=1}{\sum }}a\beta_{abc}\;{\left[A\right]}^{a}{\left[B\right]}^{b}{\left[C\right]}^{c}\\ {\left[B\right]}_{tot-calc}=\overset{nspec}{\underset{i=1}{\sum }}b\beta_{abc}\;{\left[A\right]}^{a}{\left[B\right]}^{b}{\left[C\right]}^{c}\\ {\left[C\right]}_{tot-calc}=\overset{nspec}{\underset{i=1}{\sum }}c\beta_{abc}\;{\left[A\right]}^{a}{\left[B\right]}^{b}{\left[C\right]}^{c} \end{array}$$ (5-1)

where nspec is the number of species and a, b , and c are the stoichiometric coefficients. The validity of using formation constants is, however, limited to a constant temperature. A model developed on top of these parameters cannot predict the optimized assembly sensing process under various experimental conditions. To address this limitation, we used the van’t Hoff equation to relate changes in the binding constants to changes in temperature, T , leading to estimate the change in enthalpy (ΔH) and entropy (ΔS) of a chemical interaction/reaction:

$$ln\beta =\frac{-\Delta H}{RT}+\frac{\Delta S}{R}$$ (6)

$$\begin{array}{l} {\left[A\right]}_{tot-calc}=\overset{nspec}{\underset{i=1}{\sum }}a\left(e^{\left(\frac{-\Delta H_{abc}}{RT}+\frac{\Delta S_{abc}}{R}\right)}\right)\;{\left[A\right]}^{a}{\left[B\right]}^{b}{\left[C\right]}^{c}\\ {\left[B\right]}_{tot-calc}=\overset{nspec}{\underset{i=1}{\sum }}b\left(e^{\left(\frac{-\Delta H_{abc}}{RT}+\frac{\Delta S_{abc}}{R}\right)}\right)\;{\left[A\right]}^{a}{\left[B\right]}^{b}{\left[C\right]}^{c}\\ {\left[C\right]}_{tot-calc}=\overset{nspec}{\underset{i=1}{\sum }}c\left(e^{\left(\frac{-\Delta H_{abc}}{RT}+\frac{\Delta S_{abc}}{R}\right)}\right)\;{\left[A\right]}^{a}{\left[B\right]}^{b}{\left[C\right]}^{c} \end{array}$$ (5-2)

where *R* is the ideal gas constant and equals 8.31 J/mol/K.

There are many ways of solving such a system of equations. The Newton-Raphson algorithm is usually well behaved and is relatively straightforward to implement. however, there is no guarantee for conversion and thus informative data measurements need to be introduced to deal with such cases. These computations are usually complex, and iterative algorithms have to be employed (Maeder and Neuhold, 2007). The algorithm starts with initial guesses for the free component concentrations to compute all species concentrations, and subsequently, mass conservation is checked. If there are any discrepancies, the iterative algorithm is continued. The explanations given here are insufficient for complete understanding, and more extensive definitions are beyond the scope of this study. For more details, we refer the readers to (Maeder and Neuhold, 2007). Analyzing the MATLAB codes supplied by the authors, see the end of this paper, can also assist the comprehension of the methods discussed.

### 2.2 The Newton-Gauss Algorithm

The next step is to obtain the correct ΔH and ΔS values of the combinations among the sensors and the targets using data measured at various conditions. The key point is any species in the considered interactions should exist at some minimal concentrations in the measured data. To obtain the parameters, we applied the numerical Newton-Gauss method (Bevington et al., 1993; Maeder and Neuhold, 2007) and the mathematical model of the considered sensing system to analyze the constructed data through simulations as a proxy to measured data. The measured data is always corrupted by experimental errors, instrumental shortcomings, noise, etc. The purpose of data fitting is to determine a calculated set of data, which resembles the measured data as closely as possible. The data set generated by the model is defined by the model and the thermodynamic parameters ( p ). Here, we discuss potentiometric pH titrations. The measurement is a record of the pH of the solution ( $d_{\mathrm{meas}}$ ), and the data calculated by the model is $–log\;\left(\left[H^{+}\right]\right)\;$ ( $d_{\mathrm{calc}}$ ); proton is one of the computed species concentrations. The chemical model together with the parameters allows the computation of all free species concentrations as a function of the titration. This is done by the Newton-Raphson algorithm and the results of these calculations are collected in the matrix C , which is a function of the non-linear parameters.

C=f(model, p) (7)

The non-linear parameters p require an iterative algorithm that starts with initial guesses and converges towards the optimal solution in a reasonable number of iterations and amount time. The differences between the measured and calculated data are the residuals, r :

$$r=d_{\mathrm{meas}}-d_{\mathrm{calc}}\;\left(model,p\right)$$ (8)

The measure for the quality of the fit is the sum of squares, *ssq*, which is the sum over the squares of all elements of the vector r .

$$ssq=\underset{i}{\sum }\underset{j}{\sum }r_{i,j}^{2}=f\left(model,p\right)$$ (9)

Eq. 9 demonstrates that ssq is a function of the non-linear parameters p and, of course, of the model and the data themselves that define the matrix of concentration profiles, C . These parameters are refined to minimize ssq using the Newton-Gauss method that is fast and delivers estimates for the effect of experimental noises as the standard deviations of the fitted parameters. Please see (Maeder and Neuhold, 2007) reference for more extensive explanations. The important point to notice in the fitting process is that the smaller the number of parameters, the easier the task of the fitting. Please see Section 3.2. for further discussions.

## 3 Results and Discussion

### 3.1 Plausible Mechanism of the Supramolecular Assembly

Most examples of chemical equilibria can be seen as interactions between Lewis acids and Lewis bases. In an aqueous solution, the protons are always present, and more importantly, they are also Lewis acids, which can compete with any other Lewis acid present. The protonation equilibrium can be described analogously as H+A⇌HA, and the law of mass action states that $K_{HA}=\frac{\left[HA\right]}{\left[H\right]\left[A\right]}$ .

**SCHEME 1:**
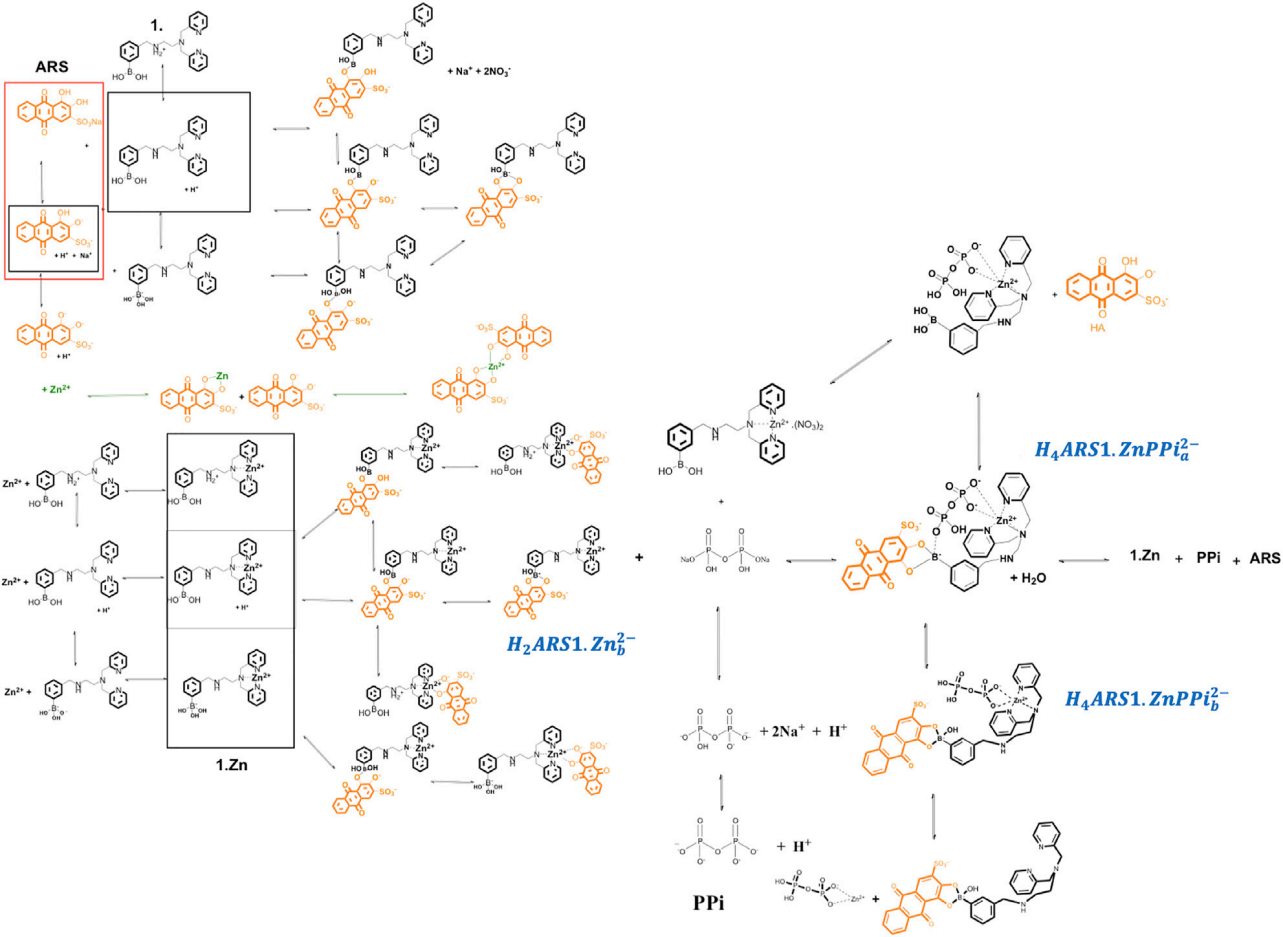
Plausible mechanism related to different interactions among 1.Zn, ARS and PPi.

The investigated supramolecular sensing system has been first introduced by Nonaka and his coworkers (Nonaka et al., 2008) to detect multi-phosphates in aqueous solutions. The synthesized receptor, DPA (DPA = dipicolylamine)-appended phenylboronic acid (**1**) in combination with Zn^2+^ (**1**·Zn) forms various assemblies with alizarin dye in a wide range of pHs. Among the existed complexes, ARS binds favorably to the coordinated zinc (II) in the Zn^2+^–DPA moiety. When PPi is added to the solution, it causes reorganization of the complex to produce an alternative boronate ester assembly. Traditionally, an indicator displacement assay is considered, and the apparent binding constants of ARS to **1**·Zn and PPi to ARS-**1**·Zn are determined. However, analytical evidence (Nonaka et al., 2008; Tomsho and Benkovic, 2012) displayed the presence of multiple intertwined equilibria shown in Scheme 1. ARS and PPi act as two- and four-protonated weak acids, $H_{2}ARS^{-}$ and $H_{4}PPi$ . Their corresponding deprotonated forms exist in a wide pH scale. **1**·Zn ( $H_{2}1.Zn^{3+}$ ) has a secondary aliphatic amine in the protonated form at lower pHs and a boronic acid moiety as a Lewis acid that exists as either a neutral trigonal-planar species at low pHs or a negatively charged tetrahedral boronate species at high pHs. Free ions $Zn^{2+}$ can also participate in various equilibrium reactions with ARS, **1**·Zn, and PPi. Further, since the solvent is water, hydrolysis dissociations can occur. PPi–ARS–1·Zn desired complex ( $H_{4}ARS1.ZnPPi_{a}^{2-}$ ) can dissociate whether to its original species- **1**·Zn, ARS, and PPi- or to PPi-**1**·Zn and ARS. The dissociation of PPi from the boronic acid moiety of **1**·Zn and eventually from the ternary complex, PPi–ARS–**1**·Zn, is another event. Also, an excess amount of **1**·Zn is capable of binding to the anion PPi. The mixture of such very complicated combinations that are not limited to this example makes it definitely impossible for the simple curve fitting to reproduce the observed progression. We used this self-assembly sensing example in the following sections as stepwise guides for researchers interested in incorporating mathematical modeling into the development of molecular sensors.

### 3.2 Separable Interactions/Reactions of ARS-1·Zn-PPi Assemblies

It is important to develop a nomenclature that uniquely describes all the species formed in solution with their appropriate equilibrium constants. Let us concentrate on the example of ARS-**1**.Zn-PPi equilibrium study in aqueous solution. After having reviewed the chemical mechanism of ARS-**1**.Zn-PPi assemblies, the next step is to determine the key chemical players and how they will interact. This step is particularly important, as it sets the stage for how complex our model will ultimately become. Using the nomenclature commonly employed in coordination chemistry, here, five components, $ARS^{3-},\;\;1^{-}$ , $PPi^{4-}$ , $H^{+}$ , and $Zn^{2+}$ , react with each other to form any complexity of species, and makes such confusing mechanism (Scheme 1). Note the expressions used: the components are the basic units that interact with each other to form the species; it is convenient to include the components in the list of species. Each of the species is formed by the appropriate number of components, and the quantitative relationship between the component and species concentrations is defined by the formation constant. The notation for equilibrium modelling of ARS-**1**.Zn-PPi example is represented in Table 1.

**TABLE 1:** Notation for equilibrium modeling of the investigated mechanism in Scheme 1.

Species	Notation					Formation constant
	ars	1	ppi	h	zn	
$\mathrm{AR}S^{-2}$	1	0	0	0	0	$\beta_{10000}=1$
1	0	1	0	0	0	$\beta_{01000}=1$
$\mathrm{PP}i^{-4}$	0	0	1	0	0	$\beta_{00100}=1$
$H^{+}$	0	0	0	1	0	$\beta_{00010}=1$
$Zn^{2+}$	0	0	0	0	1	$\beta_{00001}=1$
$\mathrm{HAR}S^{-}$	1	0	0	1	0	$\beta_{10010}=\left[\mathrm{HAR}S^{-}\right]/\left[H^{+}\right]\left[\mathrm{AR}S^{-2}\right]$
$H_{2}\mathrm{ARS}$	1	0	0	2	0	$\beta_{10020}=\left[H_{2}\mathrm{ARS}\right]/{\left[H^{+}\right]}^{2}\left[\mathrm{AR}S^{-2}\right]$
H1	0	1	0	1	0	$\beta_{01010}=\left[H1\right]/\left[H^{+}\right]\left[1\right]$
$H_{2}1^{+}$	0	1	0	2	0	$\beta_{01020}=\left[H_{2}1^{+}\right]/{\left[H^{+}\right]}^{2}\left[1\right]$
$\mathrm{HPP}i^{3-}$	0	0	1	1	0	$\beta_{00110}=\left[\mathrm{HPP}i^{3-}\right]/\left[H^{+}\right]\left[\mathrm{PP}i^{-4}\right]$
$H_{2}\mathrm{PP}i^{2-}$	0	0	1	2	0	$\beta_{00120}=\left[H_{2}\mathrm{PP}i^{2-}\right]/{\left[H^{+}\right]}^{2}\left[\mathrm{PP}i^{-4}\right]$
$H_{3}\mathrm{PP}i^{-}$	0	0	1	3	0	$\beta_{00130}=\left[H_{3}\mathrm{PP}i^{-}\right]/{\left[H^{+}\right]}^{3}\left[\mathrm{PP}i^{-4}\right]$
$H_{4}\mathrm{PPi}$	0	0	1	4	0	$\beta_{00140}=\left[H_{4}\mathrm{PPi}\right]/{\left[H^{+}\right]}^{4}\left[\mathrm{PP}i^{-4}\right]$
ZnARS	1	0	0	0	1	$\beta_{10001}=\left[\mathrm{ZnARS}\right]/\left[Zn^{2+}\right]\left[\mathrm{AR}S^{-2}\right]$
$\mathrm{ZnAR}S_{2}^{2-}$	2	0	0	0	1	$\beta_{20001}=\left[\mathrm{ZnAR}S_{2}^{2-}\right]/\left[Zn^{2+}\right]{\left[\mathrm{AR}S^{-2}\right]}^{2}$
$1Zn^{2+}$	0	1	0	0	1	$\beta_{01001}=\left[1Zn^{2+}\right]/\left[Zn^{2+}\right]\left[1\right]$
$H1Zn^{3+}$	0	1	0	1	1	$\beta_{01011}=\left[H1Zn^{3+}\right]/\left[H^{+}\right]\left[Zn^{2+}\right]\left[1\right]$
$H_{2}1Zn^{4+}$	0	1	0	2	1	$\beta_{01021}=\left[H1Zn^{3+}\right]/{\left[H^{+}\right]}^{2}\left[Zn^{2+}\right]\left[1\right]$
$\mathrm{ZnPP}i^{2-}$	0	0	1	0	1	$\beta_{00101}=\left[\mathrm{ZnPP}i^{2-}\right]/\left[Zn^{2+}\right]\left[\mathrm{PP}i^{-4}\right]$
$\mathrm{ZnOHPP}i^{3-}$	0	0	1	−1	1	$\beta_{001-11}=\left[\mathrm{ZnOHPP}i^{3-}\right]/\left[Zn^{2+}\right]\left[OH^{-}\right]\left[\mathrm{PP}i^{-4}\right]$
$\mathrm{ZnHPP}i^{-}$	0	0	1	1	1	$\beta_{00111}=\left[\mathrm{ZnHPP}i^{3-}\right]/\left[Zn^{2+}\right]\left[H^{+}\right]\left[\mathrm{PP}i^{-4}\right]$
$H_{3}1\mathrm{ZnPP}i^{-}$	0	1	1	3	1	$\beta_{01131}=\left[H_{3}1\mathrm{ZnPP}i^{-}\right]/{\left[H^{+}\right]}^{3}\left[1\right]\left[Zn^{2+}\right]\left[\mathrm{PP}i^{-4}\right]$
$H_{3}\mathrm{ARS}1^{+}$	1	1	0	3	0	$\beta_{11030}=\left[H_{3}\mathrm{ARS}1^{+}\right]/{\left[H^{+}\right]}^{3}\left[\mathrm{AR}S^{-2}\right]\left[1\right]$
$H_{2}\mathrm{ARS}1_{a}$	1	1	0	2	0	$\beta_{11020}=\left[H_{2}\mathrm{ARS}1_{a}\right]/{\left[H^{+}\right]}^{2}\left[\mathrm{AR}S^{-2}\right]\left[1\right]$
$H_{2}\mathrm{ARS}1_{b}$	1	1	0	2	0	$\beta_{11020}=\left[H_{2}\mathrm{ARS}1_{b}\right]/{\left[H^{+}\right]}^{2}\left[\mathrm{AR}S^{-2}\right]\left[1\right]$
$\mathrm{HARS}1^{-}$	1	1	0	1	0	$\beta_{11010}=\left[\mathrm{HARS}1_{b}\right]/\left[H^{+}\right]\left[\mathrm{AR}S^{-2}\right]\left[1\right]$

To have a model that can be generalized to any new complex molecular mechanism, we assumed none of the thermodynamic constants associated with the combinations/dissociations among these components are known. The first step is recognizing the interactions/reactions that can be modeled independently. In this case, some of the parameters of interest to the study can be obtained separately, which makes fitting the final model simpler by decreasing the number of unknown parameters. Accordingly, we broke the mechanism shown in Scheme 1 into ten sub mechanisms for independent investigations, Sections 3.2.1–3.2.9. Each sub-model is then followed by Eqs 3–6 to simulate the concentration profiles of the species and Eqs 7–9 to fit the thermodynamic parameters.

#### 3.2.1 Equilibria of ARS/1

$ARS^{3-}$ and $1^{-}$ act as two-protonated acids and convert to their protonated forms by changing the pH from 12 to 2. Each can be modeled and investigated by potentiometric titrations at different temperatures to compute the thermodynamic constants ( ΔH and ΔS ) of $H_{2}ARS^{-}$ , $HARS^{2-}$ , $H_{2}1^{+}$ , H1 species:

$$\begin{array}{l} H^{+}+X⇄HX\;\;\;\;\;\;\;\;\;\;\;\;\beta_{HX}=\frac{\left[HX\right]}{\left[H^{+}\right]\left[X\right]}=\frac{1}{K_{a-HX}}\\ 2H^{+}+X⇄H_{2}X\;\;\;\;\;\;\;\beta_{H_{2}X}=\frac{\left[H_{2}X\right]}{{\left[H^{+}\right]}^{2}\left[X\right]}=\frac{1}{K_{a-H_{2}X}\times K_{a-HX}}\\ X=ARS^{3-}\;or\;1^{-}\; \end{array}$$ (10)

For the components X and $H^{+}$ , the following equations define the calculated total concentrations based on the law of mass action:

$$\begin{array}{l} {\left[X\right]}_{tot-calc}=\left[X\right]+\left[HX\right]+\left[H_{2}X\right]\\ {\left[H\right]}_{tot-calc}=\left[H^{+}\right]+\left[HX\right]+2\left[H_{2}X\right]-\left[OH^{-}\right] \end{array}$$ (11)

Simulation of the species concentration profiles based on the above mathematical model is done using initial concentrations for X and proton, thermodynamic constants, and Newton-Raphson algorithm.

#### 3.2.2 Pyrophosphate Protonation Equilibria

PPi can exists in four protonated forms $HPPi^{3-},\;H_{2}PPi^{2-}$ , $H_{3}PPi^{-},$ and $H_{4}PPi$ from basic to acidic pHs.

$$\begin{array}{l} H^{+}+PPi^{4-}⇄HPPi^{3-}\;\;\;\;\;\beta_{HPPi}=\frac{\left[HPPi^{3-}\right]}{\left[H^{+}\right]\left[PPi^{4-}\right]}=\frac{1}{K_{a-HPPi}}\\ 2H^{+}+PPi^{4-}⇄H_{2}PPi^{2-}\;\;\;\;\;\;\;\beta_{H_{2}PPi}=\frac{\left[H_{2}PPi^{2-}\right]}{{\left[H^{+}\right]}^{2}\left[PPi^{4-}\right]}=\frac{1}{K_{a-H_{2}PPi}\times K_{a-HPPi}}\\ 3H^{+}+PPi^{4-}⇄H_{3}PPi^{-}\;\;\;\;\;\beta_{H_{3}PPi}=\frac{\left[H_{3}PPi^{-}\right]}{{\left[H^{+}\right]}^{3}\left[PPi^{4-}\right]}=\frac{1}{K_{a-H_{3}PPi}\times K_{a-H_{2}PPi}\times K_{a-HPPi}}\\ 4H^{+}+PPi^{4-}⇄H_{4}PPi\;\;\;\;\;\;\;\beta_{H_{4}PPi}=\frac{\left[H_{4}PPi\right]}{{\left[H^{+}\right]}^{4}\left[PPi^{4-}\right]}=\frac{1}{K_{a-H_{4}PPi}\times K_{a-H_{3}PPi}\times K_{a-H_{2}PPi}\times K_{a-HPPi}} \end{array}$$ (12)

The mass balances regarding $PPi^{4-}$ and $H^{+}$ components are as follows:

$$\begin{array}{l} {\left[PPi\right]}_{tot-calc}=\left[PPi^{4-}\right]+\left[HPPi^{3-}\right]+\left[H_{2}PPi^{2-}\right]\;+\left[H_{3}PPi^{-}\right]+\left[H_{4}PPi\right]\\ {\left[H\right]}_{tot-calc}=\left[H^{+}\right]+\left[HPPi^{3-}\right]+2\left[H_{2}PPi^{2-}\right]+3\left[H_{3}PPi^{-}\right]+4\left[H_{4}PPi\right]-\left[OH^{-}\right] \end{array}$$ (13)

#### 3.2.3 Assemblies of Zinc-Pyrophosphate

The combination of PPi with $Zn^{2+}$ can produce 1:1 complexes of ZnPPi^2−^, ZnHPPi^−^, and ZnOHPPi^3−^ (Moe and Wiest, 1977). We modeled these new products according to the following equations:

$$\begin{array}{l} Zn^{2+}+PPi^{4-}⇄ZnPPi^{2-}\;\;\;\;\;\beta_{ZnPPi}=\frac{\left[ZnPPi^{2-}\right]}{\left[Zn^{+}\right]\left[PPi^{4-}\right]}=K_{ZnPPi}\\ Zn^{2+}+PPi^{4-}+H^{+}⇄ZnHPPi^{-}\;\;\;\;\;\beta_{ZnHPPi}=\frac{\left[ZnHPPi^{-}\right]}{\left[Zn^{+}\right]\left[PPi^{4-}\right]\left[H^{+}\right]}=\frac{K_{ZnHPPi}}{K_{a-HPPi}}\\ Zn^{2+}+PPi^{4-}+OH^{-}⇄ZnOHPPi^{3-}\;\beta_{ZnOHPPi}=\frac{\left[ZnOHPPi^{3-}\right]}{{\left[Zn\right]}^{2+}\left[PPi^{4-}\right]\left[OH^{-}\right]}=K_{ZnPPi}\times K_{ZnOHPPi} \end{array}$$ (14)

The equations of conservation mass of this equilibria system used in the algorithm during the titration of pyrophosphate and zinc with HCl are:

$$\begin{array}{l} {\left[PPi\right]}_{tot-calc}=\left[PPi^{4-}\right]+\left[HPPi^{3-}\right]+\left[H_{2}PPi^{2-}\right]\;+\left[H_{3}PPi^{-}\right]+\left[H_{4}PPi\right]+\left[ZnPPi^{2-}\right]+\left[ZnHPPi^{-}\right]+\left[ZnOHPPi^{3-}\right]\\ {\left[H\right]}_{tot-calc}=\left[H^{+}\right]+\left[HPPi^{3-}\right]+2\left[H_{2}PPi^{2-}\right]+3\left[H_{3}PPi^{-}\right]+4\left[H_{4}PPi\right]+\left[ZnHPPi^{-}\right]+\left[ZnOHPPi^{3-}\right]-\left[OH^{-}\right]\\ {\left[Zn\right]}_{tot-calc}=\left[Zn^{2+}\right]+\left[ZnPPi^{2-}\right]+\left[ZnHPPi^{-}\right]+\left[ZnOHPPi^{3-}\right] \end{array}$$ (15)

#### 3.2.4 Interactions of Zinc and ARS

Zinc ions are capable of interacting with ARS in two steps to have 1:2 coordination complexes. The core structure of ARS—1,2-dihydroxyanthraquinone—serves as new lead structures for ratiometric probes for zinc ions (Kaushik et al., 2015; Zhang et al., 2007). According to the Newton-Raphson algorithm language, the reactions between $ARS^{3-}$ and $Zn^{2+}$ can be written as

$$\begin{array}{l} Zn^{2+}+ARS^{3-}⇄ZnARS^{-}\;\;\;\;\;\beta_{ZnARS}=\frac{\left[ZnARS^{-}\right]}{\left[Zn^{2+}\right]\left[ARS^{3-}\right]}=K_{ZnARS}\\ Zn^{2+}+2ARS^{3-}⇄ZnARS_{2}^{4-}\;\;\;\;\beta_{ZnARS_{2}}=\frac{\left[ZnARS_{2}^{4-}\right]}{\left[Zn^{2+}\right]{\left[ARS^{3-}\right]}^{2}}=K_{ZnARS}\;\times \;K_{ZnARS_{2}} \end{array}$$ (16)

Mass equations corresponding to ARS equilibria and its combinations with $Zn^{2+}$ are

$$\begin{array}{l} {\left[ARS\right]}_{tot-calc}=\left[ARS^{3-}\right]+\left[HARS^{2-}\right]+\left[H_{2}ARS^{-}\right]+\left[ZnARS^{2-}\right]+\left[ZnARS_{2}^{4-}\right]\\ {\left[H\right]}_{tot-calc}=\left[H^{+}\right]+\left[HARS^{2-}\right]+2\left[H_{2}ARS^{-}\right]-\left[OH^{-}\right]\\ {\left[Zn\right]}_{tot-calc}=\left[Zn^{2+}\right]+\left[ZnARS^{-}\right]+\left[ZnARS_{2}^{4-}\right] \end{array}$$ (17)

#### 3.2.5 Zinc and 1 Complexes

The three nitrogens of the DPA ligand can coordinate strongly to $Zn^{2+}$ cation, with an association constant around 10^7^ M^−1^ in water. Having three de/protonated forms in the pH 2-12 area (Eq. 10), molecule **1** can have three different combinations with $Zn^{2+}$ with probably close binding constants (Scheme 1) (Gruenwedel, 1968; O’Neil and Smith, 2006; Wong et al., 2009):

$$\begin{array}{l} Zn^{2+}+1^{-}⇄\mathrm{1.}Zn^{+}\;\;\;\;\;\beta_{1.Zn}=\frac{\left[\mathrm{1.}Zn^{+}\right]}{\left[Zn^{2+}\right]\left[1^{-}\right]}=K_{\mathrm{1.}Zn}\\ Zn^{2+}+H^{+}+1^{-}⇄H\mathrm{1.}Zn^{2+}\;\;\;\beta_{H1.Zn}=\frac{\left[H\mathrm{1.}Zn^{2+}\right]}{\left[Zn^{2+}\right]\left[H^{+}\right]\left[1^{-}\right]}=\frac{K_{H\mathrm{1.}Zn}}{K_{a-H1}}\\ Zn^{2+}+2H^{+}+1^{-}⇄H_{2}\mathrm{1.}Zn^{3+}\;\;\;\;\;\beta_{H_{2}\mathrm{1.}Zn}=\frac{\left[H_{2}\mathrm{1.}Zn^{3+}\right]}{\left[Zn^{2+}\right]{\left[H^{+}\right]}^{2}\left[{\mathrm{1.}}^{-}\right]}=\frac{K_{H_{2}\mathrm{1.}Zn}}{K_{a-H1}\times \;K_{a-H_{2}1}} \end{array}$$ (18)

The corresponding mass equations are:

$$\begin{array}{l} {\left[1\right]}_{tot-calc}=\left[1^{-}\right]+\left[H1\right]+\left[H_{2}1^{+}\right]+\left[\mathrm{1.}Zn^{+}\right]+\left[H\mathrm{1.}Zn^{2+}\right]+\left[H_{2}\mathrm{1.}Zn^{3+}\right]\\ {\left[H\right]}_{tot-calc}=\left[H^{+}\right]+\left[H1\right]+2\left[H_{2}1^{+}\right]+\left[H\mathrm{1.}Zn^{2+}\right]+2\left[H_{2}\mathrm{1.}Zn^{3+}\right]\;\;-\left[OH^{-}\right]\\ \left[Zn^{2+}\right]+\left[\mathrm{1.}Zn^{+}\right]+\left[H\mathrm{1.}Zn^{2+}\right]+\left[H_{2}\mathrm{1.}Zn^{3+}\right] \end{array}$$ (19)

#### 3.2.6 $H_{3}1\mathrm{.ZnPPi}$ Complex

The other event from Scheme 1 that can be considered separately is the interaction of $H\mathrm{1.}Zn^{2+}$ with $H_{2}PPi^{2-}$ :

$$Zn^{2+}+3H^{+}+1^{-}+PPi^{4-}⇄H_{3}\mathrm{1.}ZnPPi\;\;\;\beta_{H_{3}\mathrm{1.}ZnPPi}=\frac{\left[H_{3}\mathrm{1.}ZnPPi\right]}{\left[Zn^{2+}\right]{\left[H^{+}\right]}^{3}\left[PPi^{4-}\right]\left[1^{-}\right]}=\frac{K_{H_{3}\mathrm{1.}ZnPPi}}{K_{a-H1}\times K_{a-HPPi}\times K_{a-H_{2}PPi}}$$ (20)

Here we have four components and their related mass balances are as:

$$\begin{array}{l} {\left[1\right]}_{tot-calc}=\left[1^{-}\right]+\left[H1\right]+\left[H_{2}1^{+}\right]+\left[\mathrm{1.}Zn^{+}\right]+\left[H\mathrm{1.}Zn^{2+}\right]+\left[H_{2}\mathrm{1.}Zn^{3+}\right]+\left[H_{3}\mathrm{1.}ZnPPi\right]\\ {\left[PPi\right]}_{tot-calc}=\left[PPi^{4-}\right]+\left[HPPi^{3-}\right]+\left[H_{2}PPi^{2-}\right]+\left[H_{3}PPi^{-}\right]+\left[H_{4}PPi\right]+\left[ZnPPi^{2-}\right]+\left[ZnHPPi^{-}\right]+\left[ZnOHPPi^{3-}\right]+\left[H_{3}\mathrm{1.}ZnPPi\right]\\ {\left[H\right]}_{tot-calc}=\left[H^{+}\right]+\left[H1\right]+2\left[H_{2}1^{+}\right]+\left[H\mathrm{1.}Zn^{2+}\right]+2\left[H_{2}\mathrm{1.}Zn^{3+}\right]+3\left[H_{3}\mathrm{1.}ZnPPi\right]-\left[OH^{-}\right]\\ {\left[Zn\right]}_{tot-calc}=\left[Zn^{2+}\right]+\left[\mathrm{1.}Zn^{+}\right]+\left[H\mathrm{1.}Zn^{2+}\right]+\left[H_{2}\mathrm{1.}Zn^{3+}\right]+\left[H_{3}\mathrm{1.}ZnPPi\right] \end{array}$$ (21)

#### 3.2.7 Assemblies of ARS and 1

The next consideration was the study of the binding affinities of **1** for ARS (Tomsho and Benkovic, 2012) in solutions of varying pHs. The corresponding mass balances are

$$\begin{array}{l} {\left[1\right]}_{tot-calc}=\left[1^{-}\right]+\left[H1\right]+\left[H_{2}1^{+}\right]+\left[H_{3}ARS1_{a}^{-}\right]+\left[H_{2}ARS1_{a}^{2-}\right]+\left[H_{2}ARS1_{b}^{2-}\right]+\left[HARS1_{a}^{3-}\right]\\ {\left[ARS\right]}_{tot-calc}=\left[ARS^{3-}\right]+\left[HARS^{2-}\right]+\left[H_{2}ARS^{-}\right]+\left[H_{3}ARS1_{a}^{-}\right]+\left[H_{2}ARS1_{a}^{2-}\right]+\left[H_{2}ARS1_{b}^{2-}\right]+\left[HARS1_{a}^{3-}\right]\\ {\left[H\right]}_{tot-calc}=\left[H^{+}\right]+\left[H1\right]+2\left[H_{2}1^{+}\right]+\left[HARS^{2-}\right]+2\left[H_{2}ARS^{-}\right]+3\left[H_{3}ARS1_{a}^{-}\right]+2\left[H_{2}ARS1_{a}^{2-}\right]+2\left[H_{2}ARS1_{b}^{2-}\right]+\left[HARS1_{a}^{3-}\right]-\left[OH^{-}\right] \end{array}$$ (22)

As can be seen in Scheme 1, **1** and ARS interactions led to ten different forms of species.

#### 3.2.8 Assemblies of ARS and 1·Zn

The existence of zinc in the structure of **1** makes a new moiety for a binding competition of anions like ARS. We assumed the actual binding ability of boronic acid in **1** with ARS to form $H_{3}ARS1_{a}^{-},\;\;H_{2}ARS1_{a}^{2-},\;\;H_{2}ARS1_{b}^{2-}$ , and $HARS1_{a}^{3-}$ species is not different from that in **1**-ARS assemblies (Nonaka et al., 2008; Tomsho and Benkovic, 2012).

$$\begin{array}{l} {\left[1\right]}_{tot-calc}=\left[1^{-}\right]+\left[H1\right]+\left[H_{2}1^{+}\right]+\left[\mathrm{1.}Zn^{+}\right]+\left[H\mathrm{1.}Zn^{2+}\right]\\ +\left[H_{2}\mathrm{1.}Zn^{3+}\right]+\left[H_{3}ARS\mathrm{1.}Zn_{a}^{+}\right]+\left[H_{3}ARS\mathrm{1.}Zn_{b}^{+}\right]\\ +\left[H_{2}ARS\mathrm{1.}Zn_{a}^{2-}\right]+\left[H_{2}ARS\mathrm{1.}Zn_{b}^{2-}\right]\\ +\left[H_{2}ARS1.Zn_{c}^{2-}\right]+\left[HARS1.Zn_{a}^{3-}\;\right]\\ +\left[HARS1.Zn_{b}^{3-}\;\right]\\ {\left[ARS\right]}_{tot-calc}=\left[ARS^{3-}\right]+\left[HARS^{2-}\right]+\left[H_{2}ARS^{-}\right]+\left[ZnARS^{2-}\right]\\ +\left[ZnARS_{2}^{4-}\right]+\left[H_{3}ARS\mathrm{1.}Zn_{a}^{+}\right]\\ +\left[H_{3}ARS\mathrm{1.}Zn_{b}^{+}\right]+\left[H_{2}ARS\mathrm{1.}Zn_{a}^{2-}\right]\\ +\left[H_{2}ARS\mathrm{1.}Zn_{b}^{2-}\right]+\left[H_{2}ARS\mathrm{1.}Zn_{c}^{2-}\right]\\ +\left[HARS\mathrm{1.}Zn_{a}^{3-}\right]+\left[HARS\mathrm{1.}Zn_{b}^{3-}\right]\\ {\left[H\right]}_{tot-calc}=\left[H^{+}\right]+\left[H\mathrm{1.}\right]+2\left[H_{2}1^{+}\right]+\left[H\mathrm{1.}Zn^{2+}\right]\\ +2\left[H_{2}\mathrm{1.}Zn^{3+}\right]+\left[HARS^{2-}\right]+2\left[H_{2}ARS^{-}\right]\\ +3\left[H_{3}ARS\mathrm{1.}Zn_{a}^{+}\right]+3\left[H_{3}ARS\mathrm{1.}Zn_{b}^{+}\right]\\ +2\left[H_{2}ARS\mathrm{1.}Zn_{a}^{2-}\right]+2\left[H_{2}ARS\mathrm{1.}Zn_{b}^{2-}\right]\\ +2\left[H_{2}ARS\mathrm{1.}Zn_{c}^{2-}\right]+\left[HARS\mathrm{1.}Zn_{a}^{3-}\right]\\ +\left[HARS\mathrm{1.}Zn_{b}^{3-}\right]-\left[OH^{-}\right]\\ {\left[Zn\right]}_{tot-calc}=\left[Zn^{2+}\right]+\left[\mathrm{1.}Zn^{+}\right]+\left[H\mathrm{1.}Zn^{2+}\right]+\left[H_{2}\mathrm{1.}Zn^{3+}\right]\\ +\left[ZnARS^{2-}\right]+2\left[ZnARS_{2}^{4-}\right]+\left[H_{3}ARS1.Zn_{a}^{+}\right]\\ +\left[H_{3}ARS1.Zn_{b}^{+}\right]+\left[H_{2}ARS1.Zn_{a}^{2-}\right]\\ +\left[H_{2}ARS1.Zn_{b}^{2-}\right]+\left[H_{2}ARS1.Zn_{c}^{2-}\right]\\ +\left[HARS1.Zn_{a}^{3-}\right]+\left[HARS1.Zn_{b}^{3-}\right] \end{array}$$ (23)

According to Scheme 1, the species that are new and should be taken into account here are $H_{3}ARS1.Zn_{b}^{+}$ , $H_{2}ARS1.Zn_{c}^{2-}$ , and $HARS1.Zn_{b}^{3-}$ .

#### 3.2.9 $H_{2}\mathrm{ARS1}\mathrm{.Z}n_{b}^{\mathrm{2-}}$ - $H_{2}\mathrm{PP}i^{\mathrm{2-}}$ Interactions

The final consideration is the interactions between PPi and the formed sensor $H_{2}ARS1.Zn_{b}^{2-}$ meaning the last piece of the entire Scheme 1. Therefore, in addition to the all species noticed in previous Sections 3.2.1–3.2.8, the new species $H_{4}ARS1.ZnPPi_{a}^{2-}$ and $H_{4}ARS1.ZnPPi_{b}^{-}$ were included in our mathematical model.

$$\begin{array}{l} {\left[1\right]}_{tot-calc}=\left[1^{-}\right]+\left[H1\right]+\left[H_{2}1^{+}\right]+\left[\mathrm{1.}Zn^{+}\right]+\left[H\mathrm{1.}Zn^{2+}\right]\\ +\left[H_{2}\mathrm{1.}Zn^{3+}\right]+\left[H_{3}ARS\mathrm{1.}Zn_{a}^{+}\right]+\left[H_{3}ARS\mathrm{1.}Zn_{b}^{+}\right]\\ +\left[H_{2}ARS\mathrm{1.}Zn_{a}^{2-}\right]+\left[H_{2}ARS\mathrm{1.}Zn_{b}^{2-}\right]\\ +\left[H_{2}ARS\mathrm{1.}Zn_{c}^{2-}\right]+\left[HARS\mathrm{1.}Zn_{a}^{3-}\right]\\ +\left[HARS\mathrm{1.}Zn_{b}^{3-}\right]+\left[H_{4}ARS\mathrm{1.}ZnPPi_{a}^{2-}\right]\\ +\left[H_{4}ARS\mathrm{1.}ZnPPi_{b}^{-}\right]\\ {\left[ARS\right]}_{tot-calc}=\left[ARS^{3-}\right]+\left[HARS^{2-}\right]+\left[H_{2}ARS^{-}\right]+\left[ZnARS^{2-}\right]\\ +\left[ZnARS_{2}^{4-}\right]+\left[H_{3}ARS\mathrm{1.}Zn_{a}^{+}\right]\\ +\left[H_{3}ARS\mathrm{1.}Zn_{b}^{+}\right]+\left[H_{2}ARS\mathrm{1.}Zn_{a}^{2-}\right]\\ +\left[H_{2}ARS\mathrm{1.}Zn_{b}^{2-}\right]+\left[H_{2}ARS\mathrm{1.}Zn_{c}^{2-}\right]\\ +\left[HARS\mathrm{1.}Zn_{a}^{3-}\right]+\left[HARS\mathrm{1.}Zn_{b}^{3-}\right]\\ +\left[H_{4}ARS\mathrm{1.}ZnPPi_{a}^{2-}\right]+\left[H_{4}ARS\mathrm{1.}ZnPPi_{b}^{-}\right]\\ {\left[H\right]}_{tot-calc}=\left[H^{+}\right]+\left[H1\right]+2\left[H_{2}1^{+}\right]+\left[H\mathrm{1.}Zn^{2+}\right]\\ +2\left[H_{2}\mathrm{1.}Zn^{3+}\right]+\left[HARS^{2-}\right]+2\left[H_{2}ARS^{-}\right]\\ +3\left[H_{3}ARS\mathrm{1.}Zn_{a}^{+}\right]+3\left[H_{3}ARS\mathrm{1.}Zn_{b}^{+}\right]\\ +2\left[H_{2}ARS\mathrm{1.}Zn_{a}^{2-}\right]+2\left[H_{2}ARS\mathrm{1.}Zn_{b}^{2-}\right]\\ +2\left[H_{2}ARS\mathrm{1.}Zn_{c}^{2-}\right]+\left[HARS\mathrm{1.}Zn_{a}^{3-}\right]+\\ \left[HARS\mathrm{1.}Zn_{b}^{3-}\right]+4\left[H_{4}ARS\mathrm{1.}ZnPPi_{a}^{2-}\right]\\ +4\left[H_{4}ARS\mathrm{1.}ZnPPi_{b}^{-}\right]-\left[OH^{-}\right]\\ {\left[Zn\right]}_{tot-calc}=\left[Zn^{2+}\right]+\left[\mathrm{1.}Zn^{+}\right]+\left[H\mathrm{1.}Zn^{2+}\right]+\left[H_{2}\mathrm{1.}Zn^{3+}\right]\\ +\left[ZnARS^{2-}\right]+2\left[ZnARS_{2}^{4-}\right]+\left[H_{3}ARS1.Zn_{a}^{+}\right]\\ +\left[H_{3}ARS\mathrm{1.}Zn_{b}^{+}\right]+\left[H_{2}ARS\mathrm{1.}Zn_{a}^{2-}\right]\\ +\left[H_{2}ARS\mathrm{1.}Zn_{b}^{2-}\right]+\left[H_{2}ARS\mathrm{1.}Zn_{c}^{2-}\right]\\ +\left[HARS\mathrm{1.}Zn_{a}^{3-}\right]+\left[HARS\mathrm{1.}Zn_{b}^{3-}\right]\\ +\left[H_{4}ARS\mathrm{1.}ZnPPi_{a}^{2-}\right]+\left[H_{4}ARS\mathrm{1.}ZnPPi_{b}^{-}\right] \end{array}$$ (24)

We mathematically defined the entire interactions with any order in Scheme 1 using the concept in Table 1. Each species concentration is computed from the notation in Table 1, the formation thermodynamic constants, and the free component concentrations of H-PPi–ARS–1-Zn.

### 3.3 Guidance on the Interaction Between Mathematical Models and Experiments

With the mathematical models in place, it is now time to begin the process of using them in concert with experiments to study the molecular sensing system. Unknown thermodynamic parameters are fit by comparing model output with data, and the model itself can be assessed by predicting an unexpected experimental outcome (Banwarth-Kuhn and Sindi, 2020). To show how this step works, we simulated different data using the model and thermodynamic parameters mentioned as “used to construct data” in Tables 2, 3, 6 with noise as a proxy to measured data for each separated part of the mechanism in Scheme 1. For each recognized sub mechanism, we optimized the physicochemical conditions of the simulated potentiometric data so that any species of the considered interactions exists at some minimal concentrations in the data. For some sub-mechanisms, regardless of initial guesses of the thermodynamic parameters being far from the used ones, we reached the results with low precision and accuracy errors like equilibria of ARS/1. However, for cases that required fitting more parameters, we had to come up with initial guesses closer to the real ones, which led to a longer fitting process. One way to avoid this problem is to collect less noisy, more informative data about all species present in the equilibria. The designed experiments for the investigation of the equilibrium network consist of a series of sample solutions with different known total concentrations of the components. The samples are titrated with a strong acid (HCl) at different temperatures to produce pH data under various conditions. There is no limit to the number of possible experiments. Apart from simple cases, pH titration will always include more data than minimally required to determine a particular parameter. It increases the robustness of the analysis and help deliver statistical information about the results, such as standard deviations of the fitted parameters. Here, the hydroxide ion is defined as negative proton concentration $\left[OH^{-}\right]=-\left[H^{+}\right]$ , since in an aqueous solution, the addition of x moles of OH^-^ is equivalent to removing x moles of H^+^. Its formation constant is the equilibrium constant of water $K_{w}$ (or methanol for molecule **1**). Thermodynamic constants of water were kept fixed during all fitting processes. Of the different conditions examined, the optimized ones that lead to accurate and precise thermodynamic parameters are described in the following sections.

**TABLE 2:** Obtained thermodynamic constants for the simulated pH datasets corresponding to ARS, **1** and PPi.

	Used to construct data		Calculated		
Species	∆H (J/mole)	∆S (J/mole.K)	∆H (J/mole)	∆S (J/mole.K)	Ssq
$\mathrm{HAR}S^{2-}$	54,334	393	54,289 ± 126^a^ (−0.08%)^b^	393 ± 0.44 (−0.04%)	3.32 × 10^−4^
$H_{2}\mathrm{AR}S^{-}$	52,047	500	51,875 ± 143 (−0.33%)	499 ± 0.50 (−0.12%)	
H1	44,813	326	44,762 ± 91 (−0.11%)	326 ± 0.31 (−0.05%)	3.42 × 10^−4^
$H_{2}1^{+}$	64,878	531	64,787 ± 113 (−0.14%)	530 ± 0.39 (−0.06%)	
$\mathrm{HPP}i^{3-}$	55,206	369	55,143.9 ± 113 (−0.11%)	368 ± 0.39 (−0.06%)	8.50 × 10^−4^
$H_{2}\mathrm{PP}i^{2-}$	115,430	704	115,202 ± 145 (−0.20%)	703 ± 0.50 (−0.11%)	
$H_{3}\mathrm{PP}i^{-}$	101,380	700	100,435 ± 475 (−0.93%)	697 ± 1.63 (−0.46%)	
$H_{4}\mathrm{PPi}$	123,460	806	127,597 ± 2,693 (3.35%)	820 ± 9.02 (1.72%)	

a The standard errors associated with the fitted parameters ( $\sigma_{p}$ ) were calculated as
$$\sigma_{P}=\sigma_{R}=\sqrt{d_{i,i}}$$
where $\sigma_{R}$ represents the estimated SD of the measurement error in $d_{\mathrm{meas}}$ .
$$\sigma_{R}=\sqrt{\frac{ssq}{df}}$$
where df is the degree of freedom, which is defined as the number of experimental values m (elements of d ), subtracted by the number of optimized parameters np, df=m−np . $d_{i,i}$ is the i-th diagonal element of the inverted Hessian matrix $H^{-1}$ . Hessian matrix is the variance-covariance matrix of the parameters. The diagonal elements contain information on the parameter variances and the off-diagonal elements the covariances.
$$H^{-1}={\left(J^{t}J\right)}^{-1}$$

The Newton-Gauss algorithm for ssq minimization requires the computation of the derivatives of the residuals with respect to the parameters. These derivatives are collected in the Jacobian J .
$$J=\frac{\partial r}{\partial p}$$

Please see (Maeder and Neuhold, 2007) for more extensive explanations.
$$Accuracy=\left(\frac{P_{real}-P_{calculated}}{P_{real}}\right)\times 100$$
where $P_{real}$ are the thermodynamic parameters used to construct data and $P_{calculated}$ are the thermodynamic parameters calculated using the fitting procedure.

**TABLE 3:** Obtained affinity thermodynamic constants for the simulated pH datasets corresponding to binding affinities of $Zn^{2+}$ to PPi, ARS and **1**.

	Used to construct data		Calculated		
Species	∆H (J/mole)	∆S (J/mole.K)	∆H (J/mole)	∆S (J/mole.K)	ssq
$\mathrm{ZnPP}i^{2-}$	86,322	441	86,146 ± 189^a^ (−0.20%)^b^	439 ± 0.65 (−0.21%)	3.18 × 10^−4^
$\mathrm{ZnOHPP}i^{3-}$	99,370	326	98,711 ± 349 (−0.80%)	323 ± 1.20 (−0.20%)	
$\mathrm{ZnHPP}i^{-}$	104,390	625	104,133 ± 190 (−0.25%)	624 ± 0.65 (−0.66%)	
$\mathrm{ZnAR}S^{-}$	102,380	522	102,731 ± 549 (0.34%)	523 ± 1.89 (0.04%)	3.36 × 10^−3^
$\mathrm{ZnAR}S_{2}^{4-}$	34,127	406	34,329 ± 638 (0.59%)	406 ± 2.20 (−0.01%)	
$1.Zn^{+}$	80,299	413	80,440 ± 761 (0.18%)	413 ± 2.63 (0.12%)	3.19 × 10^−4^
$H1.Zn^{2+}$	120,450	709	120,467 ± 782 (0.01%)	709 ± 2.70 (0.01%)	
$H_{2}1.Zn^{3+}$	100,370	726	100,281 ± 1,155 (−0.09%)	725 ± 3.98 (−0.04%)	
$H_{3}1.\mathrm{ZnPPi}$	100,370	949	100,938 ± 6,993 (0.56%)	950 ± 24 (0.20%)	1.92 × 10^−3^

a The standard errors associated with the fitted parameters ( $\sigma_{p}$ ).

b Accuracy associated with the fitted parameters.

#### 3.3.1 Fitting Thermodynamic Parameters of ARS/1 Equilibria

Concentration profiles were simulated based on the equilibrium model (Eqs 1–11), different initial concentrations for X and proton, thermodynamic constants, and Newton-Raphson algorithm (Supplementary Figures S1A–D). Taking the optimized initial concentrations of the components, ${\left[E\right]}_{tot}^{0}$ = 1 mM, ${\left[H\right]}_{tot}^{0}$ = 0 ( ${\left[H\right]}_{tot}^{0}$ = 1 mM), titration is achieved by a strong acid causing change pH from ∼11 to ∼3. The total concentrations of the components at each titration point conveniently collected for both components *X* and *H* ^+^. pH changes during the titration process are used as the proxy to the measured data for fitting and acquiring equilibria information (Supplementary Figure S1E). These simulated data are collected in four augmented vectors of pH values at four different temperatures 283, 288, 293, and 298 K, [ $d_{X}^{283}$ ; $d_{X}^{288}$ ; $d_{X}^{293}$ ; $d_{X}^{288}$ ]. To more rigorously test the analysis, random errors with mean zero and SD equal to 0.002 (a realistic value for pH meters) were added to the simulated data. All measurements corresponding to each system (ARS and **1**) were thus analyzed using global analysis. The analysis process results in not only the fitted formation thermodynamic constants (the purpose) but also the matrix of concentrations for all hidden species. Supplementary Figures S1F–I displays the acquired concentration profiles for species from the fitting, which are fully matched to the simulated profiles. The augmented data at four different temperatures for each system resulted in well-defined parameters with low standard deviations (Table 2).

#### 3.3.2 Fitting Thermodynamic Parameters of Pyrophosphate Protonation Equilibria

According to the equilibria for PPi and vant’s Hoff definition, there are eight thermodynamic constants corresponding to the species to be determined. The enhanced number of unknown parameters needs finding experiment conditions that have sufficient equilibrium information. Similar to the previous section, simulation of concentration profiles based on the mathematical model (Eqs 1–9, 12–13) requires initial concentrations for the components ( PPi and H ) and thermodynamic constants (Alberty, 1969) and subsequently calls the Newton-Raphson algorithm. The pH data was generated by calculating the minus logarithm of the simulated proton concentration profiles from the model. White noise with a SD of 0.002 was added to the data. The titration of solutions with initial concentrations of ${\left[PPi\right]}_{tot}^{0}$ = 9.90 × 10^−4^ M, ${\left[H\right]}_{tot}^{0}$ = 0 at the temperatures 283, 288, 293, and 298 K with a strong acid gave pH changes from ∼11 to ∼2. Fitting the model to the constructed data at these conditions showed around 11% accuracy error for thermodynamic parameters of $H_{4}PPi$ associated with the low contribution of this species to the data (Supplementary Figures S2A–D). At 303 K and ${\left[PPi\right]}_{tot}^{0}$ = 9.90 × 10^−4^ M, and ${\left[H\right]}_{tot}^{0}$ = 2.00 × 10^−4^ M, another titration with pH variation 4.70–1.65 has been attained and added to the previous data (Supplementary Figure S2E). $H_{4}PPi$ concentration is more noticeable at the new pH window. The fitting procedure on the five augmented data [ $d_{\mathrm{PPi}}^{283}$ ; $d_{\mathrm{PPi}}^{288}$ ; $d_{\mathrm{PPi}}^{293}$ ; $d_{\mathrm{PPi}}^{288}$ ; $\;d_{\mathrm{PPi}}^{303}$ ] delivered thermodynamic parameters with low errors and the acquired concentration profiles of species (Table 2, Supplementary Figures S2F–K).

#### 3.3.3 Fitting Thermodynamic Parameters of Assemblies of Zinc-Pyrophosphate

**FIGURE 1:**
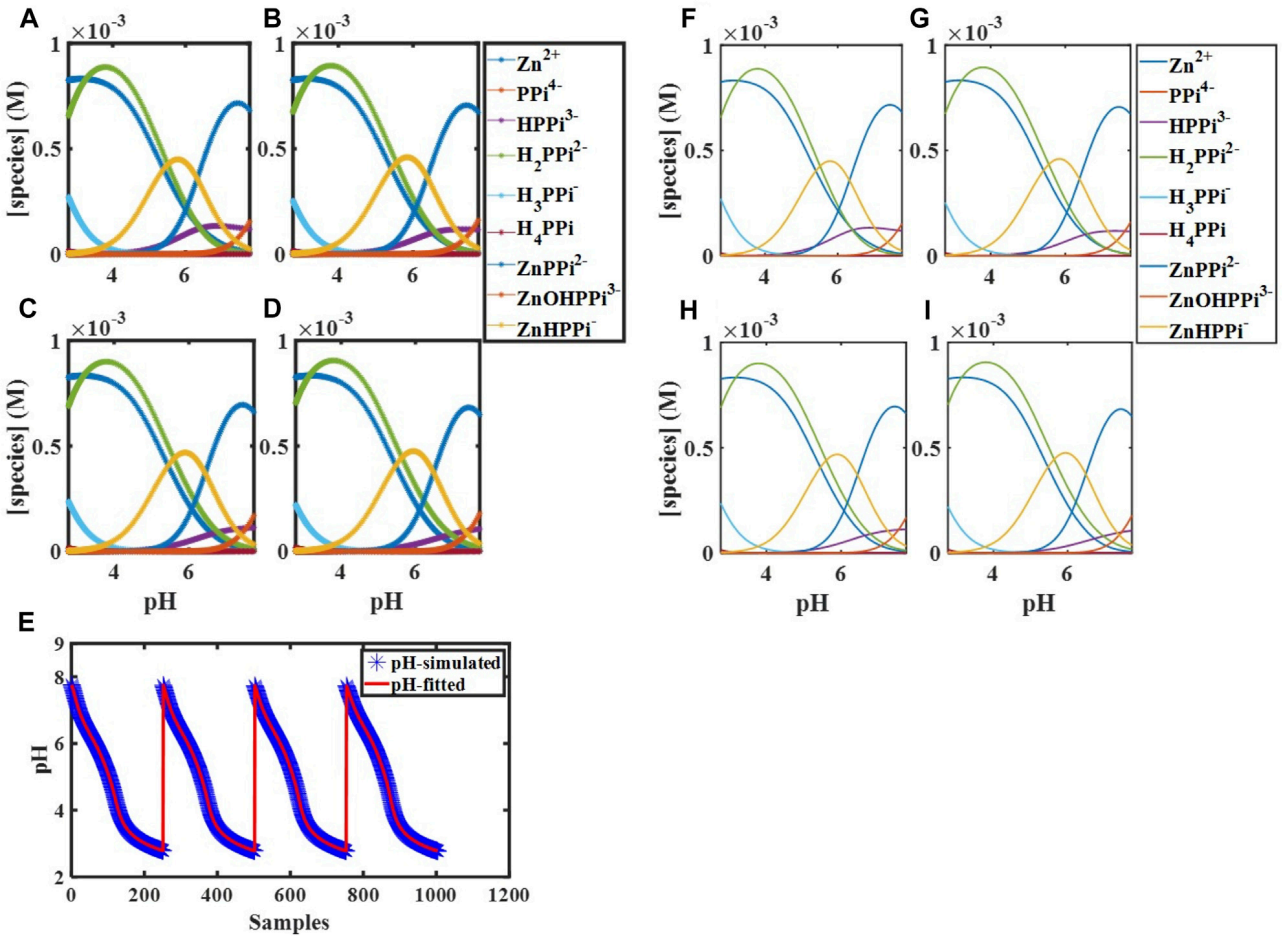
Simulated concentration profiles for ${\left[PPi\right]}_{tot}^{0}$ = 9.9 × 10^−4^ M, ${\left[Zn\right]}_{tot}^{0}\;$ = 8.7 × 10^−4^ M, and ${\left[H\right]}_{tot}^{0}$ = 0 M at **(A)** 283 K, **(B)** 288 K, **(C)** 293 K, and **(D)** 298 K; **(E)** Simulated and fitted pH for [ $d_{\mathrm{PPi}-\mathrm{Zn}}^{283}$ ; $d_{\mathrm{PPi}-\mathrm{Zn}}^{288}$ ; $d_{\mathrm{PPi}-\mathrm{Zn}}^{293}$ ; $d_{\mathrm{PPi}-\mathrm{Zn}}^{298}$ ]; Concentration profiles of species obtained using global analysis corresponding to **(F)** 283 K, **(G)** 288 K, **(H)** 293 K, and **(I)** 298 K.

Having the total concentrations of $PPi^{4-}$ , $Zn^{2+}$ , and $H^{+}$ and the thermodynamic constants (Moe and Wiest, 1977), free concentrations of species in Eqs 14, 15 are calculated *via* the model and Newton–Raphson solution. Again, pH changes during the titration process are used as the measured data for fitting and acquiring equilibria information. The concentrations of pyrophosphate and zinc ions in the potentiometric titrations were 9.9 × 10^−4^ M and 8.7 × 10^−4^ M, respectively. Higher concentrations of zinc and pyrophosphate were not considered because the precipitation of $ZnHPPi^{-}$ becomes a serious problem (Moe and Wiest, 1977). The simulated data are collected in four vectors of pH values at temperatures 283, 288, 293, and 298 K, with white noise along to SD of 0.002. pH changes at these conditions are from ∼8 to ∼3. Here, our fitting purpose was only computing zinc-pyrophosphate thermodynamic constants. Thermodynamic parameters of pyrophosphate protonation equilibria were kept known in the model obtained from Section 3.3.2. The simulated data and fitting results are given in Figures 1A–I and Table 3.

#### 3.3.4 Fitting Thermodynamic Parameters of Zinc and ARS Interactions

Concentration profiles were simulated based on the generalized equilibrium model (Eqs 16, 17) at the same temperatures and initial concentrations we used for ARS (Section 3.3.1) along with ${\left[Zn\right]}_{tot}^{0}$ = 5.00 × 10^−4^ M (Kaushik et al., 2015; Zhang et al., 2007). Next, pH changes during the titration process with white noise along to SD of 0.002 were used as the measured data [ $d_{\mathrm{ARS}-\mathrm{Zn}}^{283}$ ; $d_{\mathrm{ARS}-\mathrm{Zn}}^{288}$ ; $d_{\mathrm{ARS}-\mathrm{Zn}}^{293}$ ; $d_{\mathrm{ARS}-\mathrm{Zn}}^{298}$ ] for fitting and acquiring equilibria information of Eq. 14. Thermodynamic constants of ARS protonation are kept known in the model, since they were already computed from Eqs 10, 11 in Section 3.3.1. We collected promising fitting results in Supplementary Figures S3A–I, and Table 3.

#### 3.3.5 Fitting Thermodynamic Parameters of Zinc and 1 Complexes

Using the total concentrations of ${\left[1\right]}_{tot}$ , ${\left[H\right]}_{tot}$ , and ${\left[Zn\right]}_{tot}$ and the formation constants derived from their thermodynamic parameters (Gruenwedel, 1968; O’Neil and Smith, 2006; Wong et al., 2009), free concentrations of all species can be calculated *via* Eq. 19 and Newton–Raphson solution at each titration point. To obtain ΔH and ΔS related to each species, the temperatures used for molecule **1** titrations (Section 3.3.1) and ${\left[Zn\right]}_{tot}^{0}={\left[1\right]}_{tot}^{0}$ = 1.00 mM and ${\left[H\right]}_{tot}^{0}$ = 0 are used to generate different pH measurements based on the mentioned model with white noise (SD 0.002). The fitting results of this global analysis for this equilibrium system eventuate accurately defined parameters with the acceptable standard deviations and hidden concentrations of the involved species (Supplementary Figure S4, Table 3).

#### 3.3.6 Fitting Thermodynamic Parameters of $H_{3}1.\mathrm{ZnPPi}$ Complex

**FIGURE 2:**
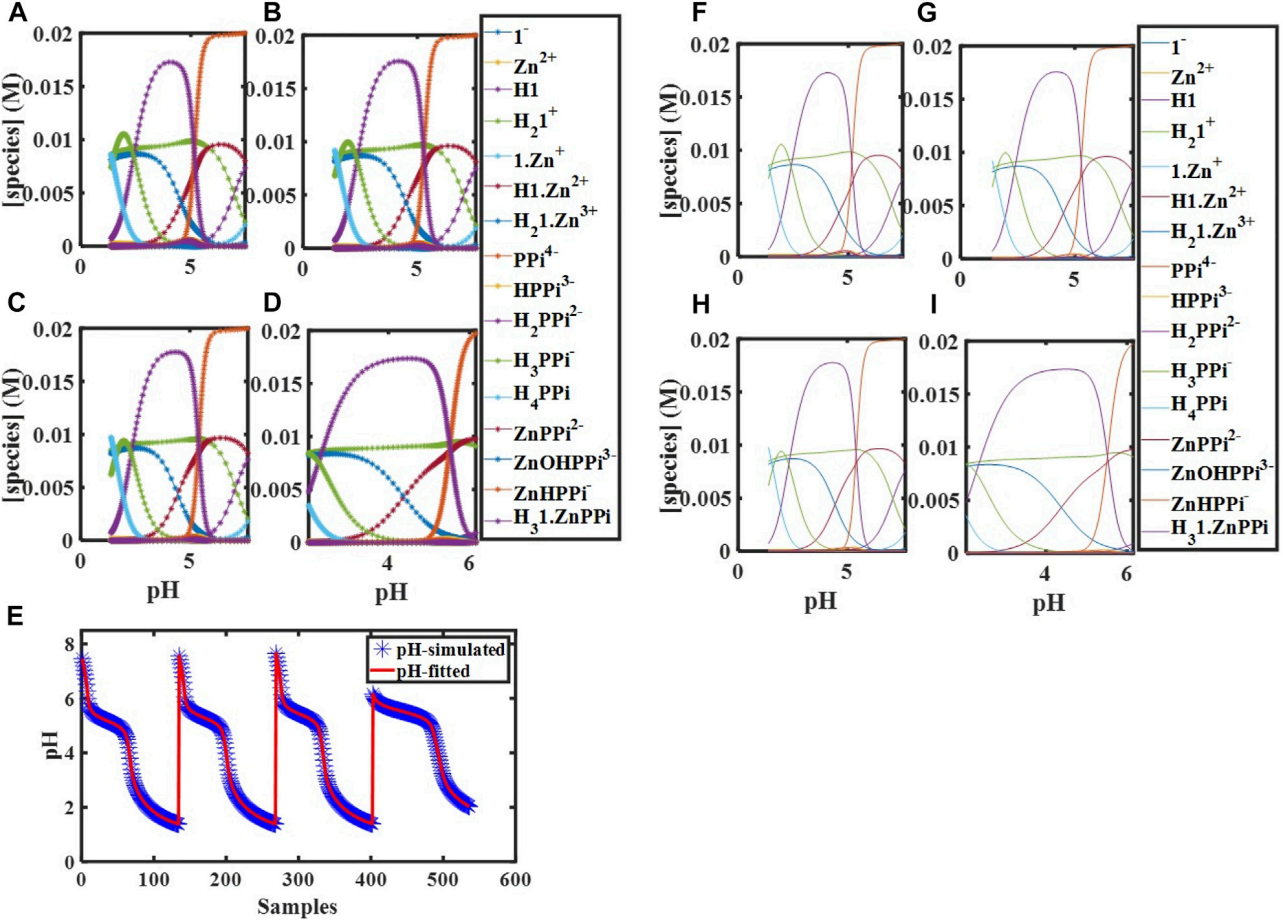
Simulated concentration profiles for ${\left[Zn\right]}_{tot}^{0}={\left[PPi\right]}_{tot}^{0}$ = 20.00 mM, ${\left[1\right]}_{tot}^{0}$ = 10.00 mM, ${\left[H\right]}_{tot}^{0}$ = 0 at **(A)** 283 K, **(B)** 288 K, **(C)** 293 K, and **(D)** for ${\left[Zn\right]}_{tot}^{0}={\left[PPi\right]}_{tot}^{0}$ = 20.00 mM, ${\left[1\right]}_{tot}^{0}$ = 10.00 mM, ${\left[H\right]}_{tot}^{0}$ = 10.00 mM at 298 K; **(E)** Simulated and fitted pH for [ $d_{1-\mathrm{Zn}-\mathrm{PPi}}^{283}$ ; $d_{1-\mathrm{Zn}-\mathrm{PPi}}^{288}$ ; $d_{1-\mathrm{Zn}-\mathrm{PPi}}^{293}$ ; $d_{1-\mathrm{Zn}-\mathrm{PPi}}^{298}$ ]; Concentration profiles of species obtained using global analysis corresponding to **(F)** 283 K, **(G)** 288 K, **(H)** 293 K, and **(I)** 298 K.

Concentration profiles were simulated based on the equilibrium model for two different initial concentrations of components, ${\left[Zn\right]}_{tot}^{0}={\left[PPi\right]}_{tot}^{0}$ = 20.00 mM, ${\left[1\right]}_{tot}^{0}$ = 10.00 mM, ${\left[H\right]}_{tot}^{0}$ = 0 at 283, 288, and 293 K showing pH changes from ∼7.50 to 1.50 and ${\left[Zn\right]}_{tot}^{0}={\left[PPi\right]}_{tot}^{0}$ = 20.00 mM, ${\left[1\right]}_{tot}^{0}$ = 10.00 mM, ${\left[H\right]}_{tot}^{0}$ = 10.00 mM at 298 K with pH changes from ∼6.00 to 2.00. According to the modeled concentration profiles (Figure 2), $H_{3}1.ZnPPi$ presents mostly in the pH range 6.00–2.00. The simulated datasets were again the pH profiles along to white noise with a SD 0.002. Here, the only unknown parameters were the thermodynamic constants corresponding to $H_{3}1.ZnPPi$ species since the rest fitted before, using Eqs 11, 13, 15, 19. The fitting procedure delivers these parameters precisely (Figure 2, Table 3).

#### 3.3.7 Fitting Thermodynamic Parameters of Assemblies of ARS and 1

The next consideration was the determination of the binding affinities of **1** to ARS in solutions of varying pHs. As can be seen in Scheme 1, ten different forms (species and components) are involved in their interactions. The thermodynamic parameters associated with $H_{2}ARS^{-}$ , $HARS^{2-}$ , $H_{2}1^{+}$ , and H1 were previously determined by separate experiment designs. The point about the rest species, $H_{2}ARS1_{a}^{2-},\;H_{2}ARS1_{b}^{2-}$ , and $HARS1_{a}^{3-}$ , is they convert each other in a triangular path. The changes in the concentrations of $H_{2}ARS1_{a}^{2-}$ and $H_{2}ARS1_{b}^{2-}$ are dependent to each other with a fully overlapped presence window of pH (Table 4). Also, the binding constants related to these species are fully correlated. Species that are formed from different ways, but, with the same components are correlated. This comes from the fact that in the final mass balance equation of $\;{\left[H\right]}_{tot-calc}$ , there is a new constant, which equals to the summation of equilibrium constants of the correlated species. Based on previous reports (Parczewski and Kateman, 1994), two completely correlated parameters cannot be computed in a chemical model because an increase or decrease of one parameter is compensated by the other. One way to solve such instances is the parameter value of one of the correlated parameters should be fixed and hence not involved in the fitting process. But, in most cases both parameter values are completely unknown and therefore, fixing one is not a viable option. In our previous study, we discovered that coupled equilibrium-kinetic mechanisms can also solve this problem (Emami et al., 2015). Here, we explored that simulating pH data at different temperatures, and initial concentrations of components and augmenting them as one dataset [ $d_{1-\mathrm{ARS}}^{278}$ ; $d_{1-\mathrm{ARS}}^{284}$ ; $d_{1-\mathrm{ARS}}^{298}$ ; $d_{1-\mathrm{AR}S_{1}}^{308}$ ; $d_{1-\mathrm{AR}S_{2}}^{308}$ ], global analysis (Table 5), allows obtaining the fully correlated binding constants (Supplementary Figure S5, Table 6). Different experiment conditions and the global analysis eliminated the full correlation between binding constants in the assemblies of ARS and 1 and led to earning their thermodynamic constants correctly. Clearly, experimental conditions have to be chosen carefully. Any species needs to exist at some minimal concentration somewhere during the titration for which the thermodynamic constants are to be determined.

**TABLE 4:** The pH presence window for the species of Scheme 1.

Species	pH	Species	pH
$\mathrm{AR}S^{2-}$	8–11.5	$\mathrm{ZnAR}S_{2}^{4-}$	5–11
$\mathrm{HAR}S^{-}$	4.5–11.5	$1.Zn^{+}$	6–11
$H_{2}\mathrm{ARS}$	4.5–7.5	$H1.Zn^{2+}$	3–11
$1^{-}$	7–11.5	$H_{2}1.Zn^{3+}$	2–8
H1	5–11	$\mathrm{ZnPP}i^{2-}$	4–11
$H_{2}1^{+}$	4–8	$\mathrm{ZnOHPP}i^{3-}$	6–11
$\mathrm{PP}i^{4-}$	7–11	$\mathrm{ZnHPP}i^{-}$	3–9
$\mathrm{HPP}i^{3-}$	4–10	$H_{3}1.\mathrm{ZnPPi}$	2–6
$H_{2}\mathrm{PP}i^{2-}$	2–9	$H_{3}\mathrm{ARS}1_{a}^{-}$	4–8
$H_{3}\mathrm{PP}i^{-}$	0.5–5	$H_{2}\mathrm{ARS}1_{a}^{2-}$	5–11
$H_{4}\mathrm{PPi}$	0.5–4	$H_{2}\mathrm{ARS}1_{b}^{2-}$	5–11
$Zn^{2+}$	1.5–10	$\mathrm{HARS}1_{a}^{3-}$	8–11.5
$\mathrm{ZnAR}S^{-}$	2–10.5	—	—

**TABLE 5:** Conditions of the simulated datasets related to ARS and **1** titration with a strong acid.

Data	Temperature (K)	${\mathrm{[ARS]}}_{\mathrm{tot}}$ (M)	${\mathrm{[H]}}_{\mathrm{tot}}$ (M)	${\mathrm{[1]}}_{\mathrm{tot}}$ (M)	${\mathrm{[H]}}_{\mathrm{titrant}}$ (M)
$d_{1-\mathrm{ARS}}^{278}$	278	1.00 × 10^−3^	2.00 × 10^−11^	1.00 × 10^−3^	18.00 × 10^−2^
$d_{1-\mathrm{ARS}}^{284}$	284				8.00 × 10^−2^
$d_{1-\mathrm{ARS}}^{298}$	298				18.00 × 10^−2^
$d_{1-\mathrm{AR}S_{1}}^{308}$	308				
$d_{1-\mathrm{AR}S_{2}}^{308}$	308		1.90 × 10^−3^		

**TABLE 6:** Obtained affinity thermodynamic constants for the simulated pH datasets corresponding to binding affinities of ARS, **1**, Zn, and PPi.

	Used to construct data		Calculated			
Species	∆H (J/mole)	∆S (J/mole.K)	∆H (J/mole)		∆S (J/mole.K)	ssq
$H_{3}\mathrm{ARS}1^{+}$	104,350	880	104,350 ± 10,883^a^ (0.00%)^b^	889 ± 37 (0.64%)		2.29 × 10^−3^
$H_{2}\mathrm{ARS}1_{a}$	71,633	691	71,633 ± 2,718 (−0.00)	691 ± 7.93 (0.001%)		
$H_{2}\mathrm{ARS}1_{b}$	37,845	571	37,845 ± 4,620 (−0.004%)	571 ± 18.17 (0.002%)		
$\mathrm{HARS}1_{a}^{-}$	28,935	351	28,935 ± 350 (0.001%)	351 ± 1.23 (0.004%)		
$H_{2}\mathrm{ARS}1.Zn_{c}^{2-}$	100,370	846	96,900 ± 51 (−1.45%)	834 ± 18.21 (−3.45%)		1.81 × 10^−3^
$H_{4}\mathrm{ARS}1.\mathrm{ZnPP}i_{a}^{2-}$	100,370	1,269	99,145 ± 2,820 (−1.22%)	1,264 ± 11.11 (−0.37%)		1.80 × 10^−3^
$H_{4}\mathrm{ARS}1.\mathrm{ZnPP}i_{b}^{-}$	130,490	1,364	128,836 ± 6,068 (−1.27%)	1,359 ± 16.97 (−0.32%)		

a The standard errors associated with the fitted parameters ( $\sigma_{p}$ ).

b Accuracy associated with the fitted parameters.

#### 3.3.8 Fitting Thermodynamic Parameters of Assemblies of ARS and 1·Zn

**FIGURE 3:**
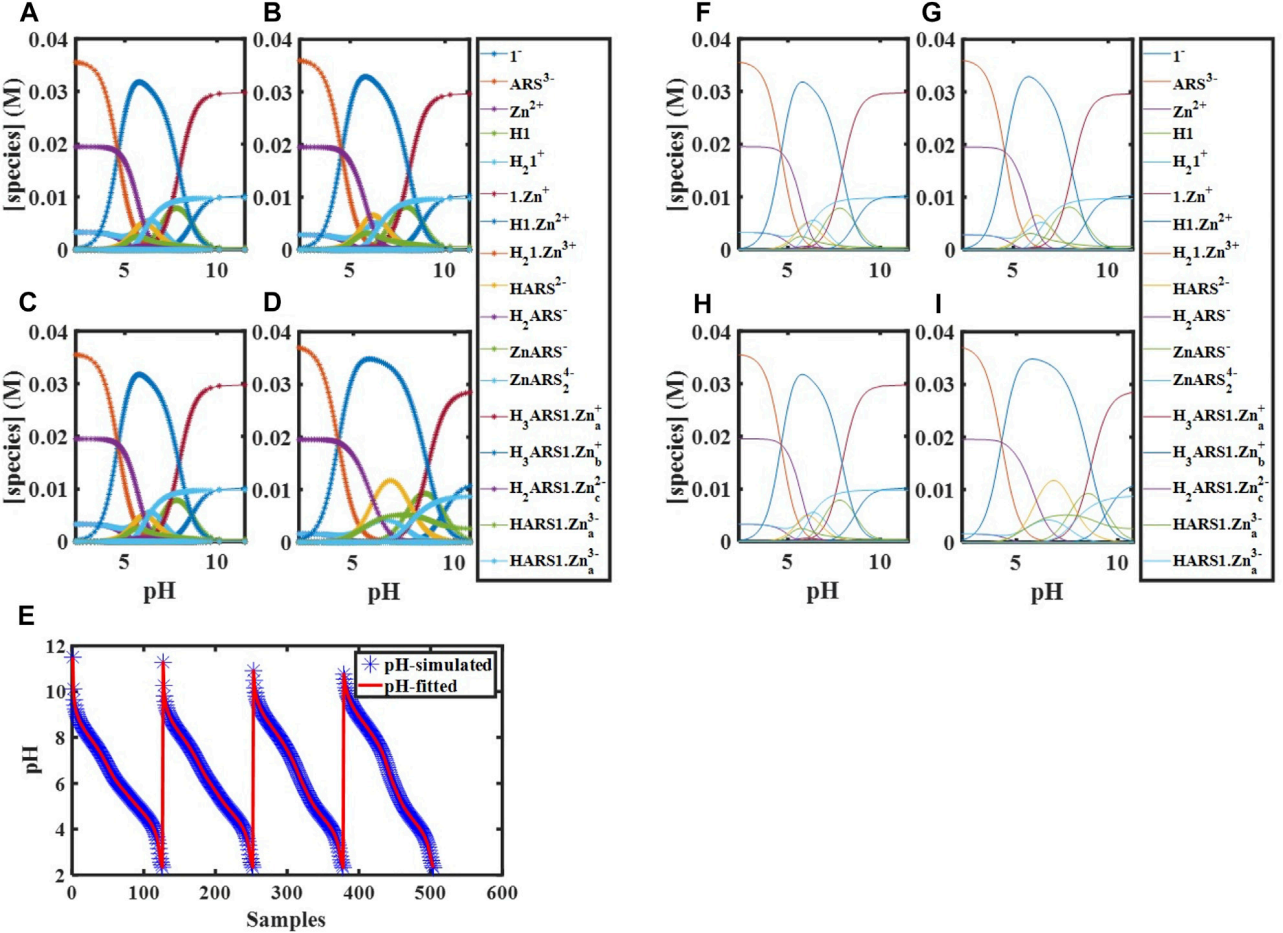
Simulated concentration profiles for ${\left[Zn\right]}_{tot}^{0}={\left[1\right]}_{tot}^{0}$ = 40.00 mM, ${\left[ARS\right]}_{tot}^{0}$ = 20.00 mM, ${\left[OH\right]}_{tot}^{0}$ = 5 × 10^−4^ M at **(A)** 278 K, **(B)** 284 K, **(C)** 298 K, and **(D)** 308 K; **(E)** Simulated and fitted pH for [ $d_{1-\mathrm{Zn}-\mathrm{ARS}}^{278}$ ; $d_{1-\mathrm{Zn}-\mathrm{ARS}}^{284}$ ; $d_{1-\mathrm{Zn}-\mathrm{ARS}}^{298}$ ; $d_{1-\mathrm{Zn}-\mathrm{ARS}}^{308}$ ]; Concentration profiles of species obtained using global analysis corresponding to **(F)** 278 K, **(G)** 284 K, **(H)** 298 K, and **(I)** 308 K.

According to Scheme 1, the species that are new and should be considered here are $H_{3}ARS1.Zn_{b}^{+}$ , $H_{2}ARS1.Zn_{c}^{2-}$ , and $HARS1.Zn_{b}^{3-}$ . We simulated several pH data at different experimental conditions for the fitting purpose. In the best conditions, using the initial concentrations of 20 mM ARS and 40 mM 1·Zn at six different temperatures with pH range 2–11, the concentrations of $H_{3}ARS1.Zn_{b}^{+}$ and $HARS1.Zn_{b}^{3-}$ do not exceed ∼1.00 × 10^−6^ and 1.00 × 10^−8^, respectively. Having these low amounts, the change in pH is less affected by these species, and so obtaining their thermodynamic parameters is impossible. The model fitting is thereby truncated by ignoring their participants, and the only considered thermodynamic parameters were related to $H_{2}ARS1.Zn_{c}^{2-}$ . At the mentioned initial concentrations and temperatures 278, 284, 298, and 308 K, ΔH and ΔS of $H_{2}ARS1.Zn_{c}^{2-}$ have been obtained with acceptable accuracy and precision (Figure 3, Table 6).

#### 3.3.9 $H_{2}\mathrm{ARS1}\mathrm{.Z}n_{b}^{\mathrm{2-}}$ **-** $H_{2}\mathrm{PP}i^{\mathrm{2-}}$ Interactions

With the thermodynamic values for the species considered before in hand, further pH titrations were performed to determine the ΔH and ΔS constants of the new species, $H_{4}ARS1.ZnPPi_{a}^{2-}$ and $H_{4}ARS1.ZnPPi_{b}^{-}$ . We simulated the concentration profiles of species stated in Eq. 24. The obtained pH values from 12 to 2 were examined to probe the associations of ARS, **1**, and Zn, with the two-protonated form of PPi. Finally, the data from conditions containing 40 mM ARS and Zn, and 20 mM **1** and PPi at four different temperatures, 278, 284, 308, and 318 K, with pH range ∼5–12 were used to determine the parameters. Simulated and calculated pH for the augmented datasets is shown in Supplementary Figure S6, Table 6. A good agreement between the simulated and calculated pHs by the model further supports our new suggestion method to obtain all equilibrium constants correctly just using the pH data. The interesting point is the possibility of target sensing by the very simple pH metric approach.

### 3.4 Predict Optimum Conditions for Pyrophosphate Monitoring Using the Developed Mathematical Model

With fit thermodynamic parameters, the mathematical model could be probed for parameter sensitivity and can predict what should happen under particular initial conditions. When we measure data, it is often only possible to experimentally observe a subset of the state variables of interest. For example, here, only the proton concentration in the form of pH can be visualized. The mathematical model can reveal the “hidden” concentrations of all other species too.

**FIGURE 4:**
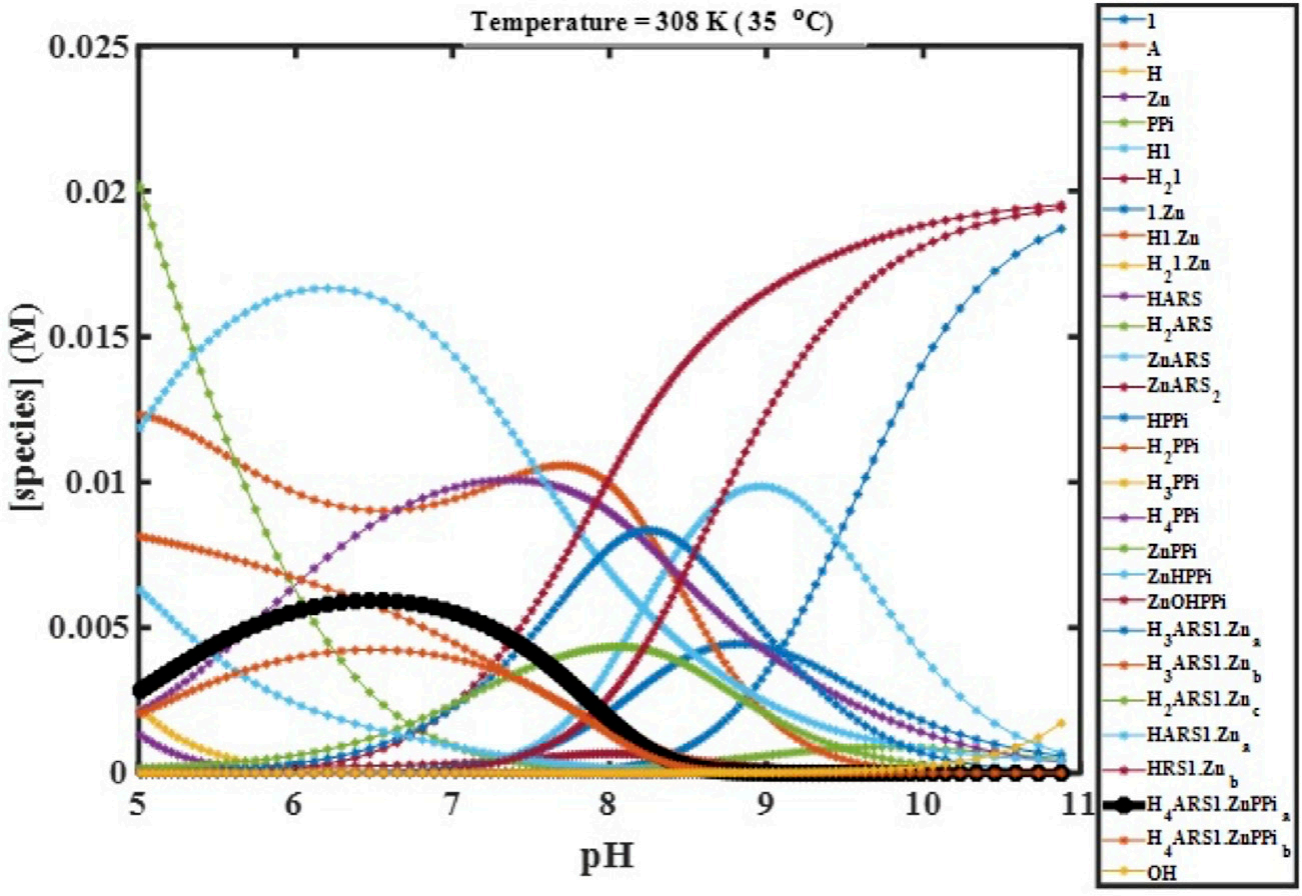
Concentration profiles of all species in the reaction mechanism (Scheme 1) at different pHs obtained using the developed model and Newton-Raphson Algorithm.

We performed computer simulations at fitted values of binding constants, different temperatures, initial concentrations, and starting pHs using the constructed mathematical model, which describes the complete molecular behavior. These simulations enabled the visualization of the experimental variables’ effects on the desired combination ( $H_{4}ARS1.ZnPPi_{a}^{2-}$ ) that produces high-intensity fluorescence response leading to PPi detection. For instance, simulation at initial concentrations 40 mM ARS, and 20 mM **1**.Zn, OH, and PPi, temperature 308 K (35°C), and pH 5–11 shows the concentration of the desired combination peaks at pH ranges from 6.2 to 7 (Figure 4). This scenario provides directions to reach the maximum possible sensitivity and selectivity of **1**.Zn-ARS assemblies towards PPi as our target by fine-tuning different experimental conditions. This mathematical model can be used to study how sensitive one output of interest is to increasing or decreasing the quantity of other factors. The mathematical model can also be used to aid in the design of new experiments. It should be pointed that the values of extracted thermodynamic parameters should be in favor of the target combination to have a practical sensing system. In case of weak interactions, tuning the extracted equilibrium constants of different combinations among the species can also enhance the desired result. For example, changing physicochemical conditions like ionic strength and/or adding/removing a component to the system can alter the values of the thermodynamic constants in favor of the desired combination in which the sensitivity increases.

## 4 Conclusion

Using mathematical modeling and the proposed stepwise experiment design, we showed how to quantitatively determine the association of the entire identified interactions among **1** Zn, ARS, PPi and their combinations. After the mathematical algorithm is written in MATLAB, we determined the thermodynamic parameters constants, ΔH and ΔS , quickly and accurately without having to rely on the assumptions that are inherent in many other common treatment methods. Various values of temperatures, initial concentrations, and starting pHs were considered to optimize the measurement conditions in the use of potentiometric assessments for thermodynamic studies. As a result, we computed all free species concentrations as a function of pH titration using the numerical Newton-Raphson algorithm, and therefore evaluation of multiple simultaneous equations is achieved. This method should be generally applicable so long as the supramolecular system is pH dependent.

Our results demonstrate the power of potentiometric titrations when used in conjunction with mathematical modeling methods to reveal details of the mechanisms of the complex fluorescent sensors used widely in biological chemistry. The significance of this subject can be very much enhanced by the possibility of easily describing mathematically the behavior of complex systems and fine-tuning them for different applications. The very important point in gaining potentiometric data is the pH changes related to the presence region of each species should have adequate titration points. Based on these insights, we highlight how the extraction of binding data from a network of equilibria using mathematical modeling can produce optimum conditions for real-time oligophosphate monitoring. General speaking, the results represent a significant step forward in elucidating the chemistry of complex fluorescent probes and provide a paradigm for future work in this area.

## Data Availability

The original contributions presented in the study are included in the article/Supplementary Material, further inquiries can be directed to the corresponding author.
