# Supplementary material for: Mathematical Modeling of a Supramolecular Assembly for Pyrophosphate Sensing
Source: Front Chem. 2021 Dec 21;9:759714. doi: 10.3389/fchem.2021.759714 (PMC8724255; doi:10.3389/fchem.2021.759714)
Supplement: Supplementary file 1 [file DataSheet1.PDF]

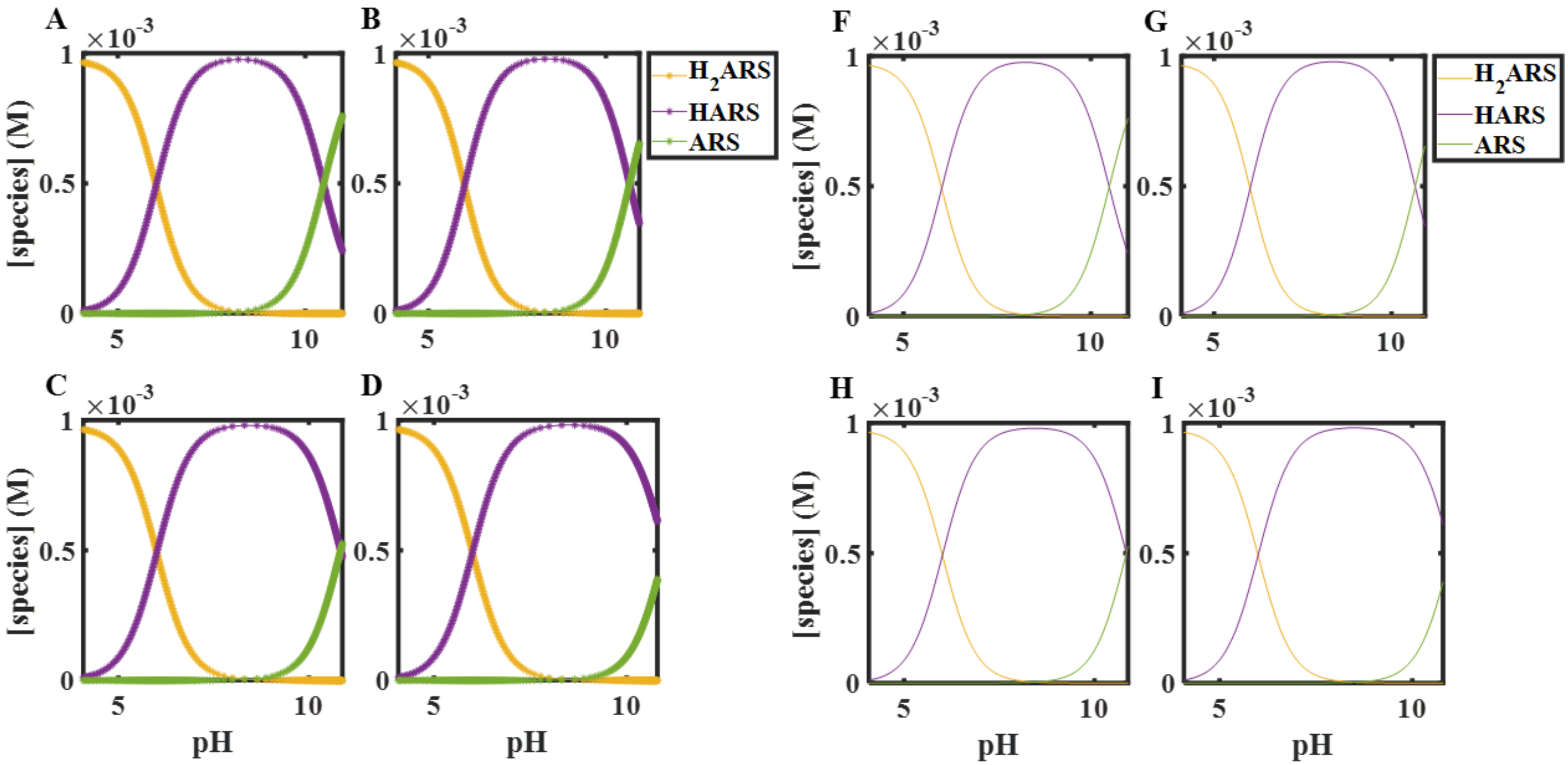

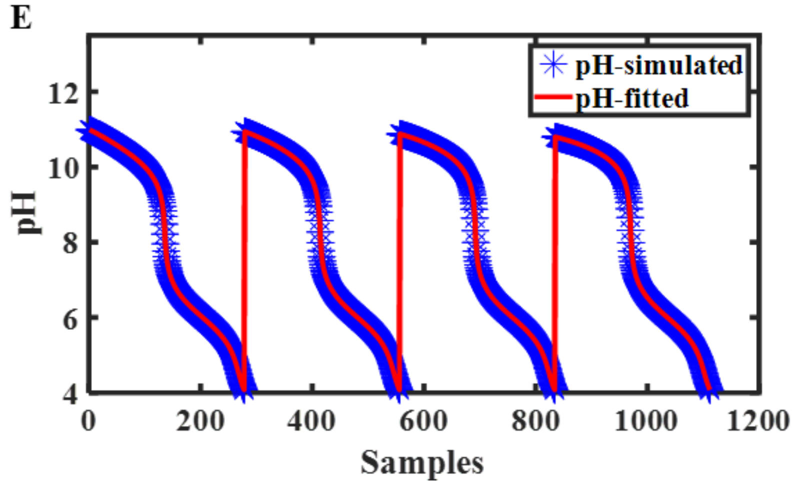

Figure S1. Simulated concentration profiles for $[ARS]^0_{tot}$ =1 mM, $[H]^0_{tot}$ =0 ($[OH]^0_{tot}$ =1 mM) at **(A)** 283 K, **(B)** 288 K, **(C)** 293 K, and **(D)** 298 K; **(E)** Simulated and fitted pH for $[\boldsymbol{d}^{283}_{ARS}; \boldsymbol{d}^{288}_{ARS}; \boldsymbol{d}^{293}_{ARS}; \boldsymbol{d}^{288}_{ARS}]$; Concentration profiles of species obtained using global analysis corresponding to **(F)** 283 K, **(G)** 288 K, **(H)** 293 K, and **(I)** 298 K.

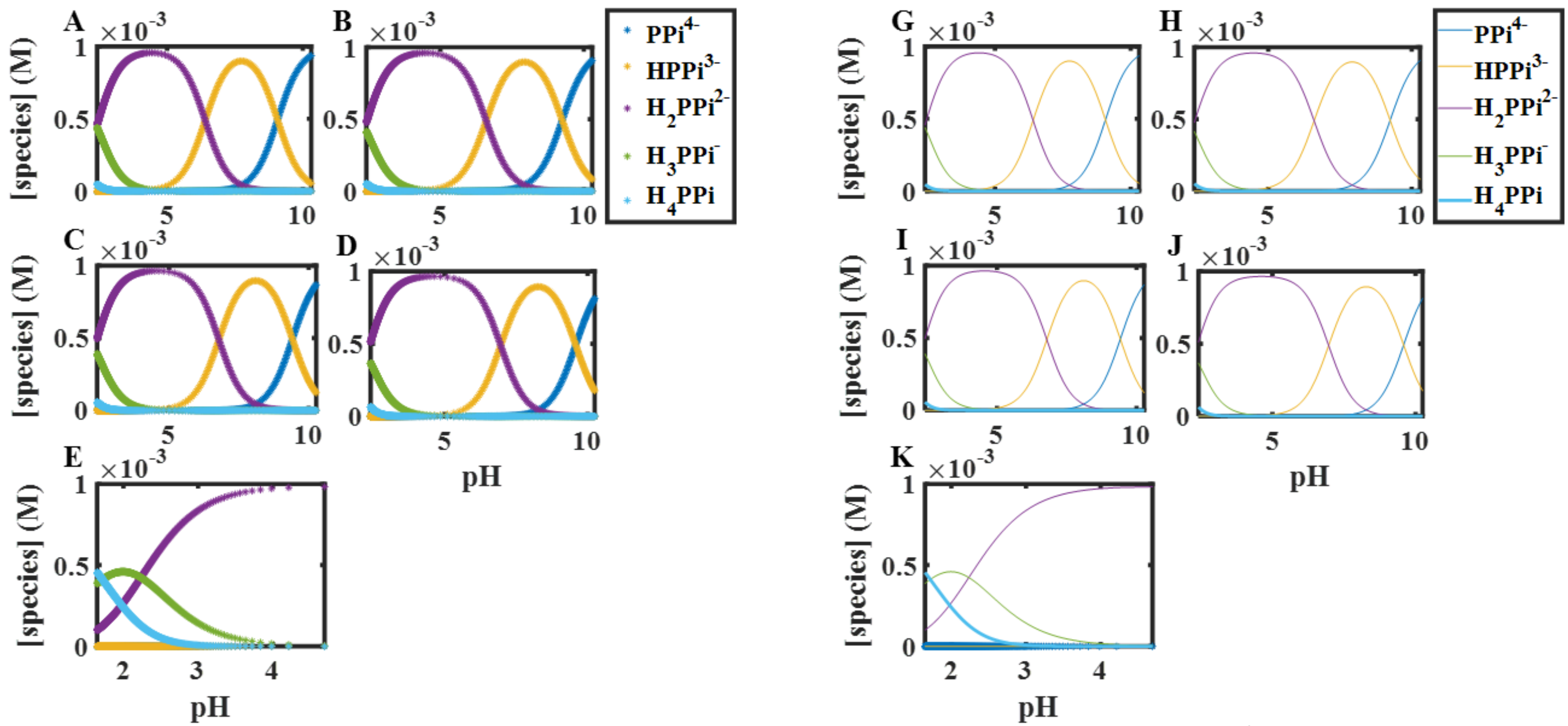

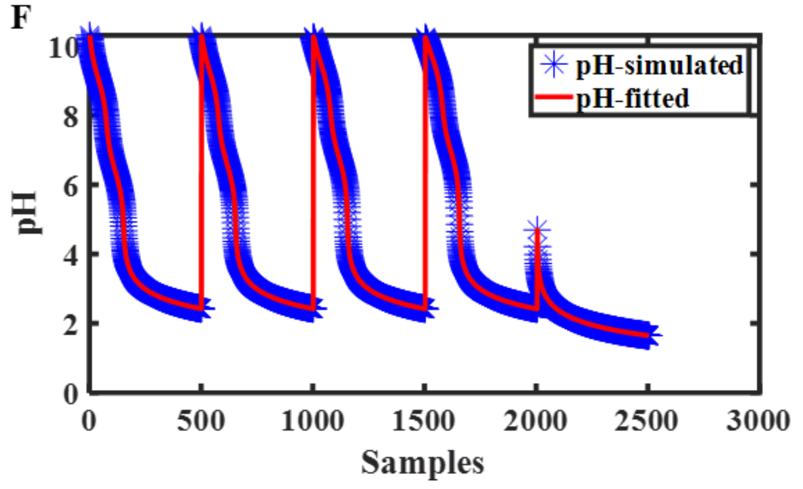

Figure S2. Simulated concentration profiles for $[PPi]^0_{tot}$ =9.90×10^{-4} M, $[H]^0_{tot}$ =0 at **(A)** 283 K, **(B)** 288 K, **(C)** 293 K, and **(D)** 298 K; Simulated concentration profiles for $[PPi]^0_{tot}$ =9.90×10^{-4} M, and $[H]^0_{tot}$ =2.00×10^{-4} M at **(E)** 303 K; **(F)** Simulated and fitted pH for [$\boldsymbol{d^{283}_{PPi}}$; $\boldsymbol{d^{288}_{PPi}}$; $\boldsymbol{d^{293}_{PPi}}$; $\boldsymbol{d^{288}_{PPi}}$; $\boldsymbol{d^{303}_{PPi}}$]; Concentration profiles of species obtained using global analysis corresponding to **(G)** 283 K, **(H)** 288 K, **(I)** 293 K, **(J)** 298 K, and **(K)** 303 K.

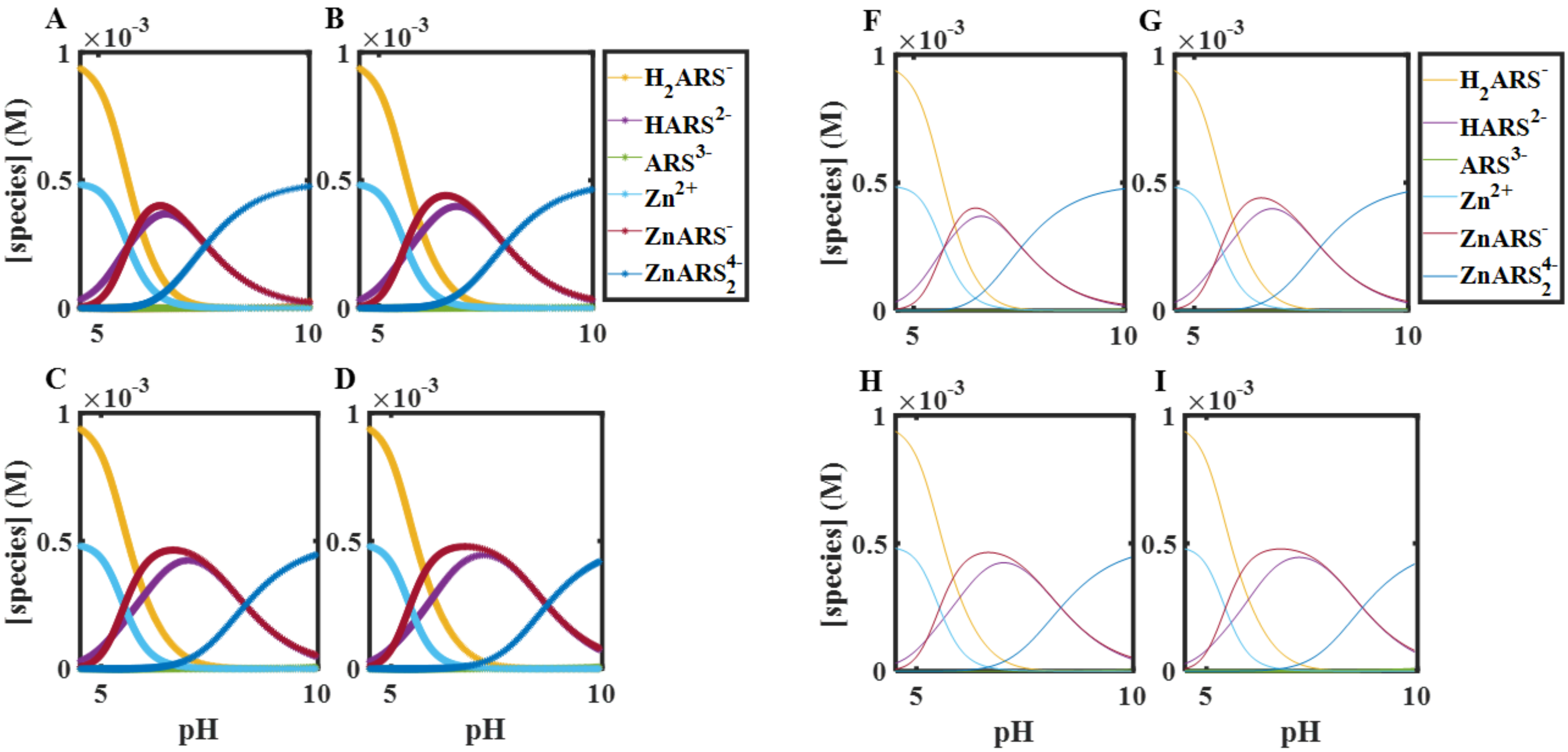

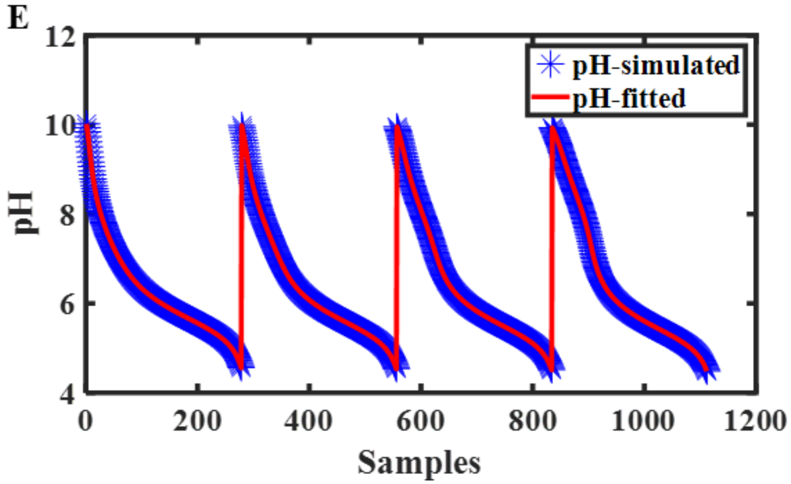

Figure S3. Simulated concentration profiles for $[ARS]^0_{tot}$ =1 mM, $[H]^0_{tot}$ =0 ($[OH]^0_{tot}$ =1 mM), and $[Zn]^0_{tot}$ =5.00×10^{-4} M at **(A)** 283 K, **(B)** 288 K, **(C)** 293 K, and **(D)** 298 K; **(E)** Simulated and fitted pH for $[\boldsymbol{d}^{283}_{ARS-Zn};\ \boldsymbol{d}^{288}_{ARS-Zn};\ \boldsymbol{d}^{293}_{ARS-Zn};\ \boldsymbol{d}^{298}_{ARS-Zn}]$; Concentration profiles of species obtained using global analysis corresponding to **(F)** 283 K, **(G)** 288 K, **(H)** 293 K, and **(I)** 298 K.

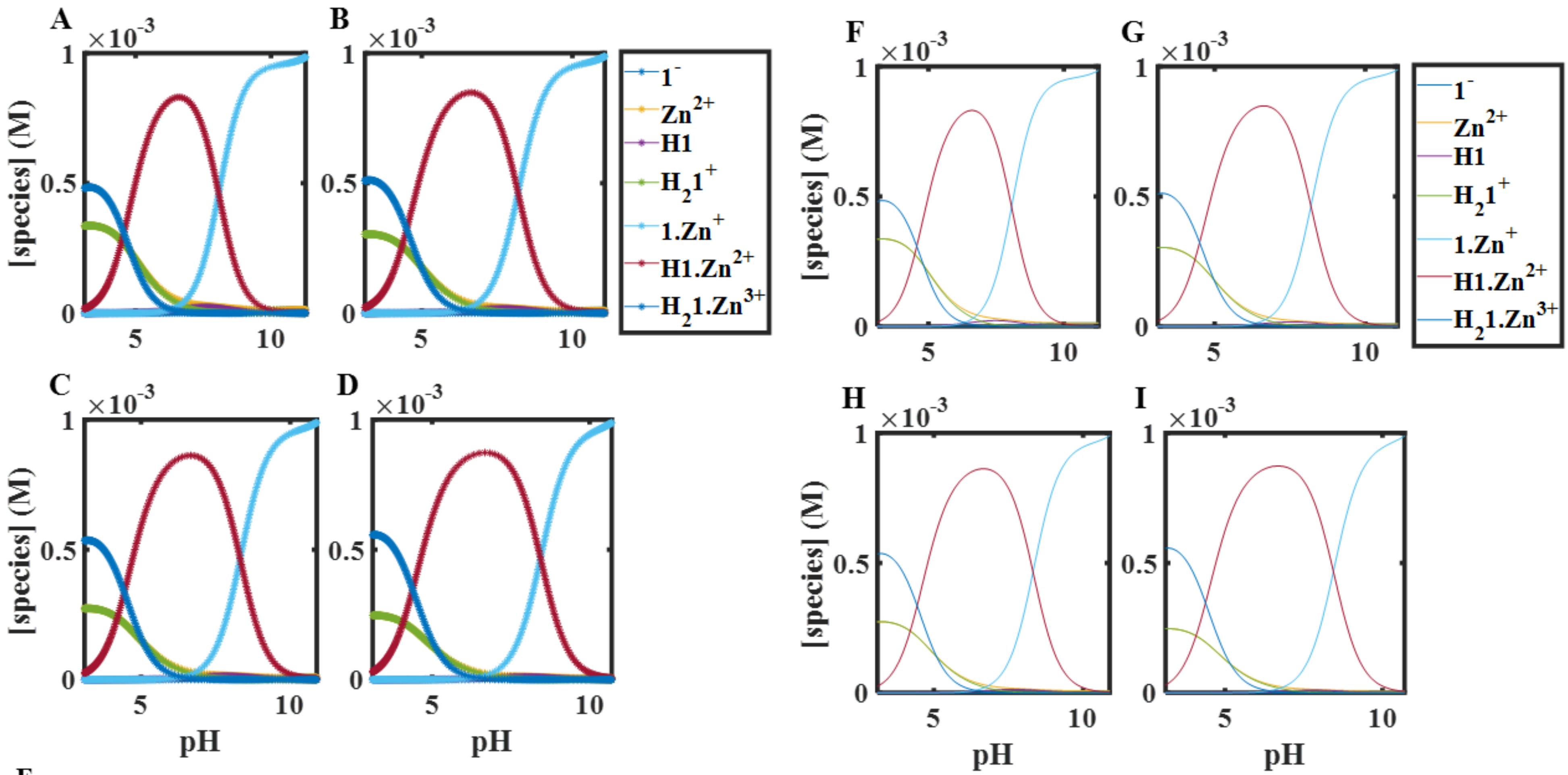

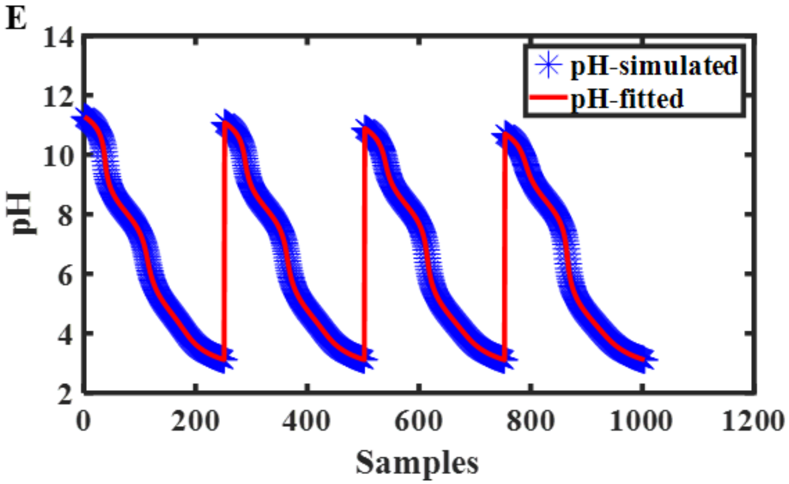

Figure S4. Simulated concentration profiles for $[Zn]^0_{tot} = [1]^0_{tot}$ =1 mM, $[H]^0_{tot}$ =0 at **(A)** 283 K, **(B)** 288 K, **(C)** 293 K, and **(D)** 298 K; **(E)** Simulated and fitted pH for $[\boldsymbol{d}^{283}_{1-Zn}; \boldsymbol{d}^{288}_{1-Zn}; \boldsymbol{d}^{293}_{1-Zn}; \boldsymbol{d}^{298}_{1-Zn}]$; Concentration profiles of species obtained using global analysis corresponding to **(F)** 283 K, **(G)** 288 K, **(H)** 293 K, and **(I)** 298 K.

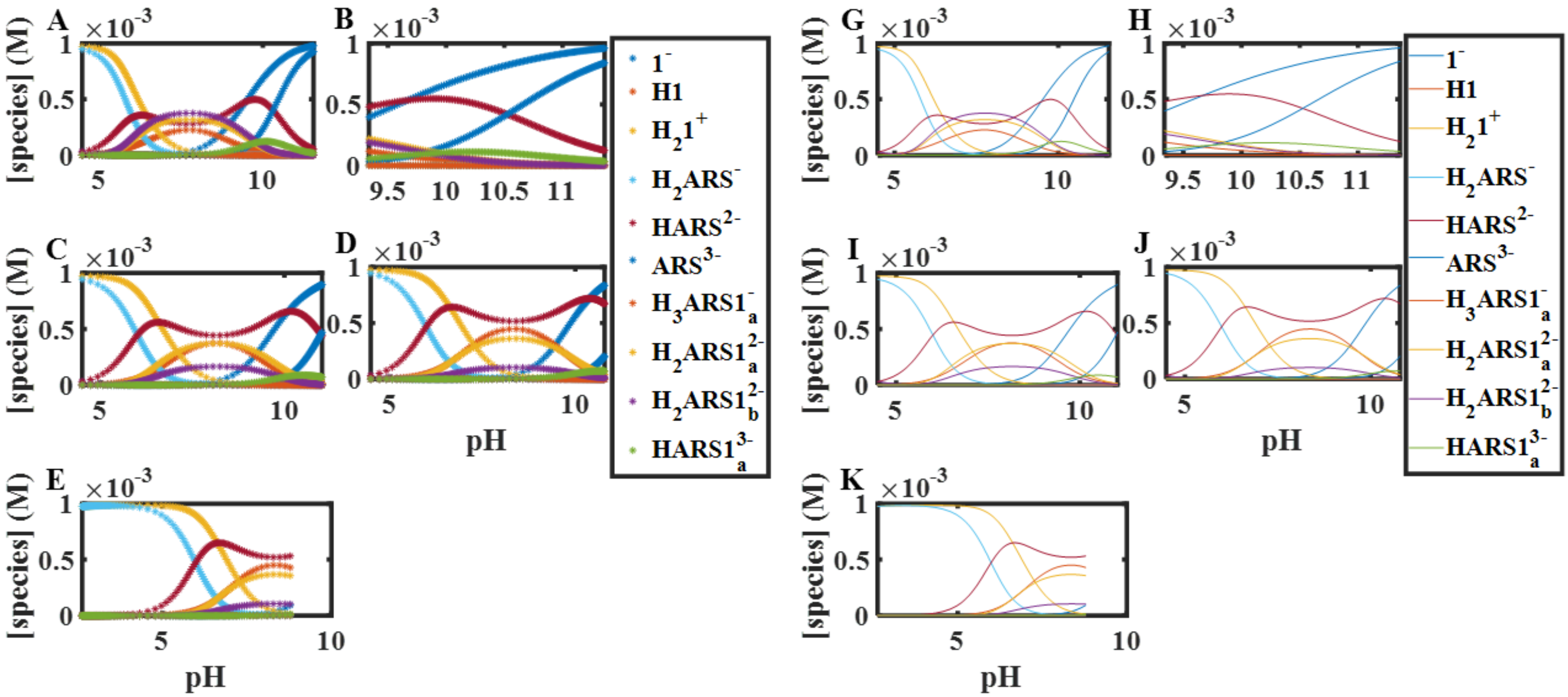

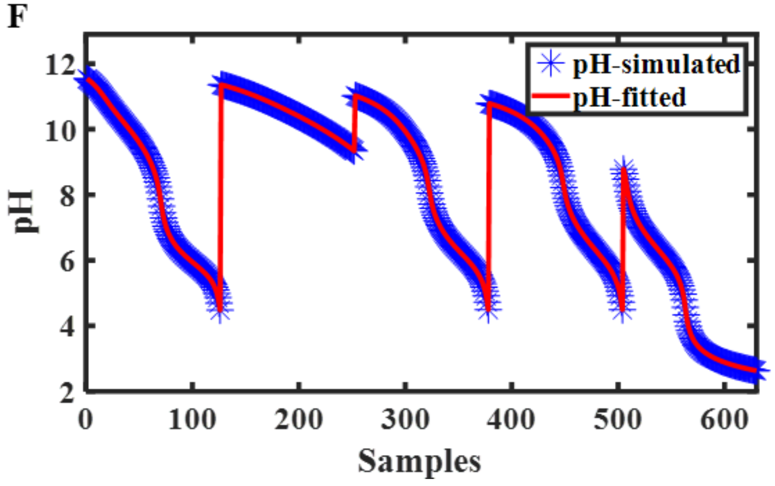

Figure S5. Simulated concentration profiles for **(A)** $[ARS]^0_{tot}$ = 1.00 mM, $[\mathbf{1}]^0_{tot}$= 1.00 mM, and $[H]^0_{tot}$= 2.00×10^{-11} at 278 K, **(B)** $[ARS]^0_{tot}$ = 1.00 mM, $[\mathbf{1}]^0_{tot}$= 1.00 mM, and $[H]^0_{tot}$= 2.00×10^{-11} at 284 K, **(C)** $[ARS]^0_{tot}$ = 1.00 mM, $[\mathbf{1}]^0_{tot}$= 1.00 mM, and $[H]^0_{tot}$= 2.00×10^{-11} at 298 K, **(D)** $[ARS]^0_{tot}$ = 1.00 mM, $[\mathbf{1}]^0_{tot}$= 1.00 mM, and $[H]^0_{tot}$= 2.00×10^{-11} at 308 K, and **(E)** $[ARS]^0_{tot}$ = 1.00 mM, $[\mathbf{1}]^0_{tot}$= 1.00 mM, and $[H]^0_{tot}$= 1.90×10^{-3} at 308 K; **(F)** Simulated and fitted pH for [$\boldsymbol{d^{278}_{1-ARS}}$; $\boldsymbol{d^{284}_{1-ARS}}$; $\boldsymbol{d^{298}_{1-ARS}}$; $\boldsymbol{d^{308}_{1-ARS_1}}$;$\boldsymbol{d^{308}_{1-ARS_2}}$]; Concentration profiles of species obtained using global analysis corresponding to **(G)** 278 K, **(H)** 284 K, **(I)** 298 K, **(J)** $\boldsymbol{1-ARS_1}$ 308 K, and **(K)** $\boldsymbol{1-ARS_2}$ 308 K.

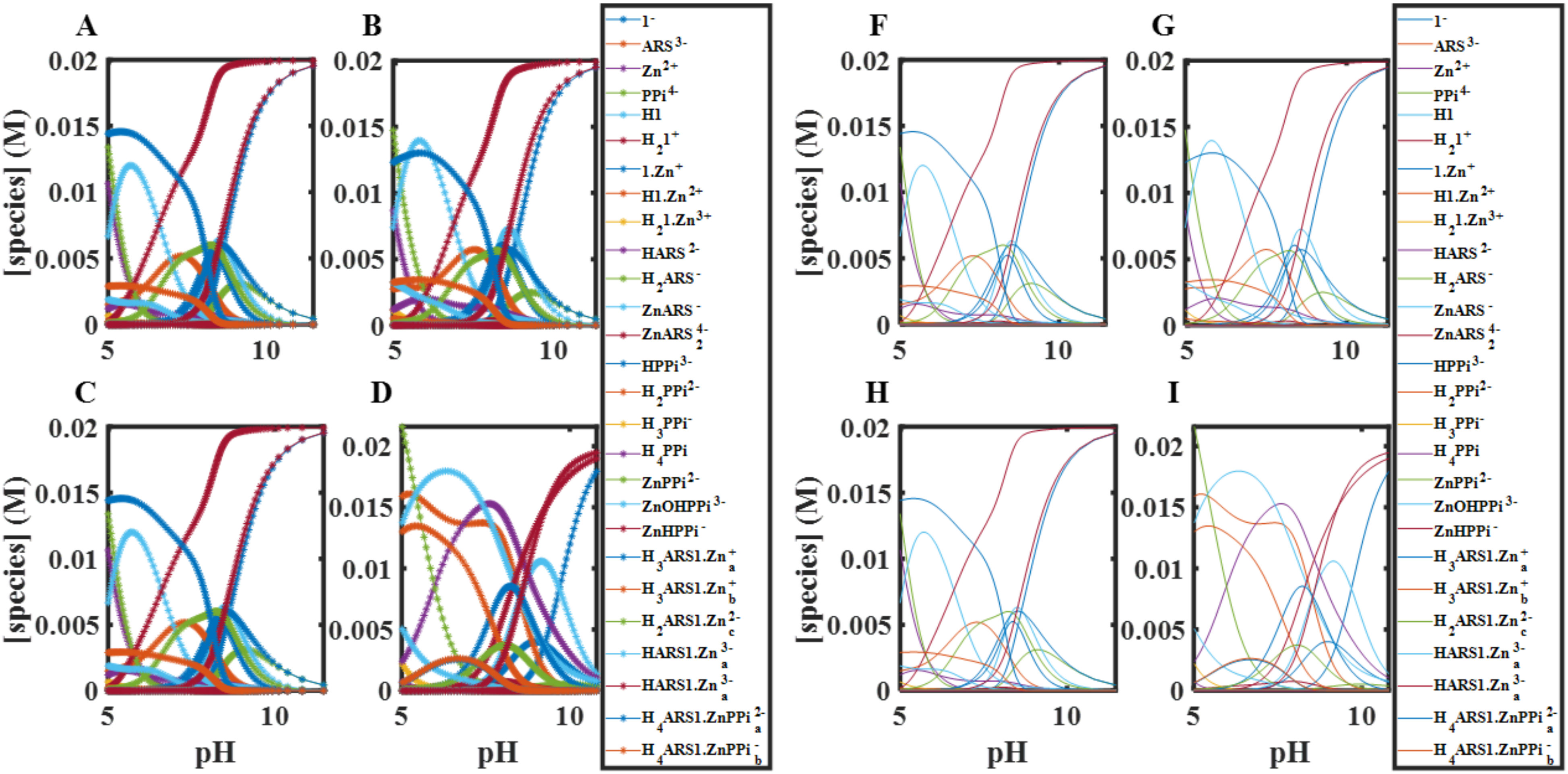

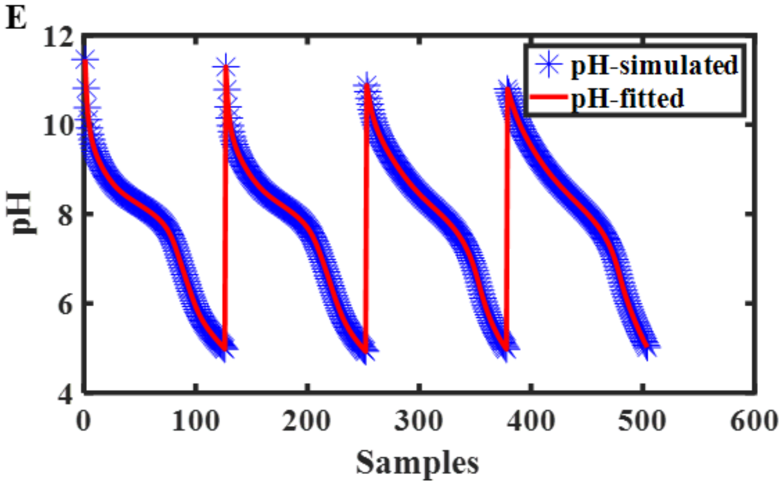

Figure S6. Simulated concentration profiles for $[Zn]_{tot}^{0} = [\mathbf{1}]_{tot}^{0}$=40.00 mM, $[ARS]_{tot}^{0}$=$[PPi]_{tot}^{0}$=20.00 mM, $[OH]_{tot}^{0}$= 2×10^{-2} M at **(A)** 278 K, **(B)** 284 K, **(C)** 308 K, and **(D)** 318 K; **(E)** Simulated and fitted pH for $[\boldsymbol{d}_{\mathbf{1-Zn-ARS-PPi}}^{\mathbf{278}};\ \boldsymbol{d}_{\mathbf{1-Zn-ARS-PPi}}^{\mathbf{284}};\ \boldsymbol{d}_{\mathbf{1-Zn-ARS-PPi}}^{\mathbf{308}};\ \boldsymbol{d}_{\mathbf{1-Zn-ARS-PPi}}^{\mathbf{318}}]$; Concentration profiles of species obtained using global analysis corresponding to **(F)** 278 K, **(G)** 284 K, **(H)** 308 K, and **(I)** 318 .
